# Multi-Omics Reveals the Impact of Domestic Wastewater Input on the Dissolved Organic Carbon Pool and Microbial Community in the Qiantang River Estuary

**DOI:** 10.3390/microorganisms14061282

**Published:** 2026-06-05

**Authors:** Yun-Fei Cao, Yi-Ru Wang, Pei-Xin Zheng, Xing-Chen Wang, Lin Xu, Cong Sun

**Affiliations:** 1 College of Life Sciences and Medicine, Zhejiang Sci-Tech University, Hangzhou 310018, China; 2023220902006@mails.zstu.edu.cn (Y.-F.C.); yr_1414@163.com (Y.-R.W.); 18320663742@163.com (P.-X.Z.); linxu@zstu.edu.cn (L.X.); 2 Zhejiang Engineering Research Center for the Development Technology of Medicinal and Edible Homologous Health Food, Shaoxing Biomedical Research Institute of Zhejiang Sci-Tech University Co., Ltd., Shaoxing 312075, China; xc684107@outlook.com

**Keywords:** domestic wastewater, recalcitrant dissolved organic carbon (RDOC), microbial carbon pump (MCP), multi-omics, estuarine carbon sink

## Abstract

Estuarine ecosystems face intense anthropogenic pressures, yet systematic research on how domestic wastewater influences the dissolved organic carbon (DOC) pool via microbial community regulation remains limited. In this study, we conducted a microcosm experiment simulating wastewater input into the Qiantang River and integrated multi-omics (16S rRNA sequencing, metagenomics, metatranscriptomics, and FT-ICR MS) to elucidate the mechanism. Results showed that: (1) Wastewater input increased initial DOC and changed its degradation pattern: slower decay but higher removal. (2) Compared to the control, the wastewater-amended group exhibited a decreased fluorescence intensity contribution of carboxyl-rich alicyclic molecule (CRAM)-like compounds, indicating reduced chemical stability of recalcitrant DOC (RDOC). (3) Wastewater drove directional microbial succession from catabolic-dominant taxa (e.g., *Comamonas*, *Citrobacter*) to anabolic-dominant taxa (e.g., *Reyranella*), shifting metabolism from pollutant degradation to endogenous synthesis, thereby lowering the system’s efficiency in forming stable RDOC. (4) Multi-omics revealed a “stimulation-balance” functional response: early activation of xenobiotic degradation and signal transduction (day 2), followed by a shift to anabolic metabolism (day 28). This functional transition, driven by microbial succession, ultimately reduced RDOC stability. Our findings reveal that wastewater reshapes the microbial carbon pump, providing a theoretical basis for assessing estuarine carbon sink responses to pollution control measures.

## 1. Introduction

Estuarine regions serve as critical conduits for transporting terrestrial materials into the ocean. They function not only as key hubs for the exchange of matter and energy between rivers and seas but also face superimposed disturbances from intensive human activities [1]. In recent years, accelerated coastal urbanization, agricultural intensification, and industrialization have caused substantial inputs of nitrogen, phosphorus, and various organic pollutants into estuaries via pathways such as domestic wastewater discharge [2]. These anthropogenic discharges alter nutrient composition and disrupt the stoichiometric balance of carbon, nitrogen, and phosphorus in the water, which may profoundly reshape the pathways and efficiency of microbial carbon cycling [3,4]. Against this backdrop, in-depth analysis of estuarine carbon cycling processes under anthropogenic interference—particularly the mechanisms underlying the formation, transformation, and stability maintenance of RDOC—is of significant importance for scientifically assessing the ecological functions and carbon sequestration potential of estuaries, as well as for predicting their responses in the context of global change [5,6].

Dissolved organic carbon (DOC) is one of the primary carriers of carbon cycling in aquatic environments. Based on differences in bioavailability, DOC can be categorized into labile and recalcitrant components [5,7]. Among these, RDOC refers to the DOC fraction characterized by strong resistance to degradation, capable of persisting in water bodies over long time scales (from centuries to millennia) [7]. It is noteworthy that systematic differences exist between RDOC in coastal estuaries and the open ocean regarding their sources, formation mechanisms, and fates: RDOC in coastal estuaries primarily originates from terrestrial inputs, undergoes rapid microbial transformation under intense physical mixing and steep chemical gradients, exhibits relatively lower recalcitrance, and mainly serves as a temporary carbon reservoir and a conduit for transport to the open ocean. In contrast, RDOC in the open ocean is predominantly derived from phytoplankton-derived organic matter, undergoes long-term and repeated microbial reworking, possesses highly complex structures and extreme recalcitrance, and is ultimately sequestered in the deep ocean for millennia, constituting a core component of the millennial-scale carbon sink [7,8]. The microbial carbon pump theory emphasizes that microbial activities, including complex metabolic processes such as synthesis, modification, transformation, and polymerization, are the core drivers shaping the formation and properties of RDOC [8,9].The sources, composition, and transformation pathways of RDOC in estuarine areas are more complex, influenced not only by marine microbial activities but also closely linked to the composition of terrestrial inputs, rapid changes in physicochemical conditions of the water body, and the consequent succession in microbial community structure and function [10].

The dynamic and heterogeneous nature of estuarine environments presents unique challenges for RDOC research. High-load organic matter from terrestrial inputs (including both natural organic matter and anthropogenic pollutants) may serve directly as precursors for RDOC, or indirectly influence RDOC generation by altering microbial metabolic strategies [11]. For example, the input of exogenous pollutants (such as polycyclic aromatic hydrocarbons, plasticizers, etc.) may induce microorganisms to activate specific degradation and detoxification pathways [12,13]. The metabolic intermediates may contribute to the RDOC pool, but the chemical structures and stability of these products may be altered. Beyond the intrinsic functional potential of microorganisms, environmental factors such as the carbon-to-nitrogen ratio (C/N), dissolved oxygen, salinity, pH, and temperature [14] can also indirectly influence the formation pathways and final properties of RDOC by regulating microbial metabolic efficiency and community succession. Among these, the carbon-to-nitrogen stoichiometric ratio (C/N) is recognized as a key factor regulating microbial metabolic strategies and element allocation [15]. It may affect microbial nitrogen acquisition and utilization efficiency, shift the balance between anabolic and catabolic metabolism, and thereby determine the chemical composition and stability of RDOC.

However, existing research on RDOC formation mechanisms has largely focused on the open ocean or oligotrophic waters. There remains a lack of systematic understanding of the microbial regulatory mechanisms governing RDOC in estuarine ecosystems, which are subject to intense terrestrial inputs, pronounced environmental gradients, and frequent anthropogenic disturbances [16]. Specifically, three major gaps persist in the current literature: (1) most previous studies have focused on open-ocean or oligotrophic waters, with insufficient attention to highly disturbed estuarine systems; (2) previous work has predominantly used single-omics approaches (e.g., 16S rRNA sequencing or DOM characterization alone), making it difficult to establish causal links from microbial community structure to RDOC molecular properties; and (3) the mechanistic understanding of how changes in the C/N ratio regulate microbial metabolic strategies to affect RDOC stability remains particularly limited. Based on the above understanding, this study selects the Qiantang River estuary, which is significantly impacted by human activities, as the research subject. Using an indoor microcosm experiment to simulate the altered C/N condition induced by domestic wastewater input, and integrating multi-omics techniques including 16S rRNA gene amplicon sequencing, metagenomics and metatranscriptomics, as well as Fourier transform ion cyclotron resonance mass spectrometry (FT-ICR MS), we comprehensively applied these methods to systematically investigate the impact mechanisms of domestic wastewater input on the molecular composition and stability of DOC, as well as on microbial community structure and functional gene expression. This study aims to reveal: (1) how domestic wastewater input alters the concentration, molecular composition, and chemical stability of DOC in water; (2) the key microbial taxa driving these DOC dynamics and their response patterns to wastewater stress; and (3) the core functional genes and pathways regulating DOC metabolism within these key taxa. The findings will elucidate the influence mechanisms of wastewater input on estuarine DOC from multiple dimensions—“organic molecular characteristics—community structure–functional genes”—providing a scientific basis for pollution control and carbon sink management in estuarine regions.

## 2. Materials and Methods

### 2.1. Study Site and Sample Collection

Surface water (approximately 60 L) was collected from the Zhutoujiao Dam site (120°18′44.07″ E, 30°17′19.00″ N) in the Qiantang River Estuary. Samples were immediately stored in a cooler at 4 °C and transported to the laboratory within 6 h. The water was then filtered through 0.7 μm glass fiber filters (Whatman, Cytiva, Hauppauge, NY, USA) (pre-combusted at 450 °C) to remove most plankton and suspended particles, yielding approximately 50 L of pre-treated water for subsequent microcosm experiments.

To ensure the accuracy of DOC measurement results, all apparatus that comes into direct contact with samples during the experiment must undergo a strict carbon-removal pretreatment procedure. The specific pretreatment protocol is as follows: First, all types of experimental apparatus, including glass fiber filters (GF/F), brown glass bottles, pipettes, filtration setups, and sample vials—are sequentially rinsed three times each with tap water, reverse osmosis (RO) water (purified water with most ions and organics removed), and ultrapure (MQ) water (deionized water with a resistivity of 18.2 MΩ·cm, typically produced by a Milli-Q system). This stepwise rinsing progressively removes particulate matter and water-soluble impurities adhering to the surfaces of the apparatus [17]. Subsequently, the initially cleaned apparatus is fully immersed in a 2 mol/L hydrochloric acid solution and allowed to incubate at room temperature for 48 h. During this period, the items are periodically agitated to ensure thorough contact between the acid and all surfaces, thereby completely dissolving and removing any potential inorganic carbon residues [17]. After soaking, the apparatus is repeatedly rinsed with copious amounts of MQ water until the effluent reaches a neutral pH, followed by air-drying in a clean environment [17]. To further eliminate any possible residual organic carbon contaminants, glassware capable of withstanding high temperatures (such as glass bottles and sample vials) undergoes high-temperature ashing: the items are placed in a muffle furnace and combusted at 450 °C for 4 h, allowing any remaining organic carbon components to fully decompose and ash under the high temperature [18].

### 2.2. Preparation of Simulated Domestic Wastewater and Microcosm Setup

A 4 L stock solution of simulated domestic wastewater was prepared with the following specific composition and concentrations: The carbon source consisted of sucrose (4.8 g), a mixture of volatile fatty acids (acetic, propionic, butyric, and valeric acids, 0.48 mL each, total 1.92 mL), and humic acid (0.08 g), designed to reflect the co-occurrence of readily and poorly degradable organic carbon in wastewater. The nitrogen source included ammonium chloride (6 g), urea (0.18 g), and peptone (6 g), encompassing common forms of inorganic and organic nitrogen. Phosphorus was added in the form of dipotassium hydrogen phosphate (0.84 g) and potassium dihydrogen phosphate (0.66 g). Furthermore, sodium dodecyl benzene sulfonate (0.08 g) was added to simulate surfactants commonly found in domestic wastewater [19]. All components were dissolved in ultrapure water, and sodium bicarbonate (12 g) was used as a buffer to stabilize the system’s pH within the range of 7.0 ± 0.2, approximating the pH environment of actual wastewater [19].

Based on the measured total organic carbon (TOC) and total nitrogen (TN) of the initial Qiantang River water and the simulated domestic wastewater described above, the two were mixed in a specific ratio (the simulated domestic wastewater: Qiantang River water = 5:100, *v*/*v*) to prepare an experimental group (denoted as Group S) with a carbon-to-nitrogen (C/N) ratio of approximately 6, simulating the water condition after receiving domestic wastewater input. Simultaneously, the original Qiantang River water without simulated wastewater addition (C/N ≈ 3) served as the control group (denoted as Group Q). For each group, a total of 24 L of water was used. This volume was evenly distributed into twelve brown glass bottles, resulting in 2 L per bottle. The bottle mouths were covered with breathable sealing film, and the bottles were placed in a constant-temperature incubator for cultivation under dark conditions (temperature controlled at the in situ average water temperature of 20 ± 1 °C) [20]. A destructive sampling strategy was employed: each of the 11 time points used a separate bottle, which was sacrificed after sampling. Thus, no repeated sampling from the same bottle occurred, and the headspace-to-liquid ratio remained unchanged throughout the incubation. To maintain water homogeneity and facilitate gas exchange, the bottles were gently shaken daily and briefly vented (by loosening the caps) [20,21]. During the cultivation period, samples were collected at 11 time points (days 0, 0.5, 1, 2, 3, 4, 5, 7, 14, 21, and 28) for the measurement of key environmental parameters, including total organic carbon (TOC), total nitrogen (TN), total phosphorus (TP), and chemical oxygen demand (COD) [21]. Based on a preliminary biodegradation experiment, DOC concentrations declined rapidly within the first two days and then stabilized by day 28. Therefore, day 0 (baseline), day 2 (early stimulus phase), and day 28 (stable phase) were selected for multi-omics analysis. To further investigate the dynamic changes in microbial community structure, functional potential, and metabolic activity, representative time-point samples—including the initial water sample (day 0), as well as samples from Groups Q and S collected on day 2 (early response phase) and day 28 (late stable phase)—were selected for multi-omics analysis. These samples were subjected to FT-ICR MS analysis, 16S rRNA gene amplicon sequencing, metagenomic sequencing, and metatranscriptomic sequencing to systematically reveal the profound impacts of domestic wastewater input on microbial ecological processes and organic matter transformation.

### 2.3. Determination of Environmental Parameters and Data Analysis

The measurement of all environmental parameters followed standard methods, with each sample analyzed in triplicate to ensure data reliability. Specifically, total nitrogen (TN) was determined using an elemental analyzer (Elementar UNICUBE, Rhine, Germany). DOC was measured by a TOC analyzer (Shimadzu TOC-L, Kyoto, Japan) based on the non-purgeable organic carbon (NPOC) method [22]. Total phosphorus (TP) was quantified using an ultraviolet spectrophotometer (Shanghai Youke 752N, Shanghai, China) following the molybdenum-antimony anti-spectrophotometric method. Chemical oxygen demand (COD) was measured using a water quality analyzer (HACH DR3900, Shanghai, China). The systematic statistical analysis and visualization were performed using Origin 2021 software.

### 2.4. Solid-Phase Extraction

The water samples were first retrieved from frozen storage, thawed at room temperature, and equilibrated to approximately 25 °C, then transferred to a clean beaker. High-purity hydrochloric acid (12 mol/L, Sinopharm Chemical Reagent Co., Ltd., Shanghai, China) was used to slowly adjust the sample pH to 2.0 ± 0.1, with gentle stirring to ensure uniform acidification. After acidification, the samples were stored in a 4 °C refrigerator in the dark to prevent photodegradation or microbial activity, generally for no more than 48 h before extraction [23]. Prior to solid-phase extraction (SPE), the SPE cartridges were conditioned [23]. Each cartridge was fixed on a stand and sequentially rinsed with three column volumes of methanol followed by three column volumes of acidified ultrapure water (pH = 2) at a flow rate of approximately 1 mL/min. This step activated the stationary phase, removed potential residual impurities, and kept it wet for subsequent loading [24]. To meet the FT-ICR MS requirement for an extracted sample concentration of 50–100 mg/L, the DOC concentration of each water sample was measured in advance to determine the required loading volume [24]. Based on the DOC value and the target concentration range, the required sample loading volume was calculated precisely to ensure the extracted concentration met the instrument’s detection requirements [25]. The accurately measured volume of water sample was then slowly passed through the conditioned SPE cartridge under gravity at a stable flow rate, allowing for the adsorption and enrichment of organic matter onto the stationary phase [25]. After loading, the cartridge was rinsed with three column volumes of acidified ultrapure water (pH = 2) to remove residual inorganic salts and some hydrophilic impurities. Immediately following rinsing, any remaining moisture in the cartridge was completely removed using a gentle stream of nitrogen. This step prevented water from interfering with the subsequent elution efficiency and ensured effective recovery of organic compounds. Once the cartridge was thoroughly dried, the adsorbed organics were eluted using a suitable volume of high-purity methanol at a relatively slow flow rate [26]. The entire eluate was collected into an Agilent sample vial, ensuring a final collection volume greater than 1 mL [26]. After elution, the sample was immediately stored at −20 °C in the dark to prevent degradation or volatilization of the organic compounds until FT-ICR MS analysis.

### 2.5. FT-ICR MS Analysis

The solid-phase extracted samples were sent to the Analytical and Testing Center of Beijing Petroleum University for high-resolution molecular characterization using a Bruker SolariX XR 9.4 T Fourier (Billerica, MA, USA) transform ion cyclotron resonance mass spectrometer (FT-ICR MS). The acquired raw mass spectra were calibrated and subjected to quality control (HRMS Viewer) before being exported as lists containing *m/z* (mass-to-charge ratio) and peak intensity information. Mass spectra were acquired in negative ion mode using electrospray ionization (ESI). The mass range was *m*/*z* 100–1000, with a mass accuracy of <0.5 ppm (internal calibration) and a resolution of >1,500,000 at *m*/*z* 400. Molecular formula assignment was constrained to C (1–100), H (1–200), O (1–50), N (0–5), S (0–2), P (0–2), with a maximum mass error of ±1 ppm.

Van Krevelen diagrams were generated in Origin 2021 software by plotting the hydrogen-to-carbon atomic ratio (H/C) against the oxygen-to-carbon atomic ratio (O/C) for each assigned molecular formula. The normalized peak intensity of each formula was used as a third dimension represented by a color gradient from red (highest relative abundance) to green to yellow (lowest relative abundance), providing a visual representation of the distribution and relative intensity of different organic compound classes in the chemical space [27]. Following the molecular classification system established by Xu et al., molecular formulas with H/C ratios between 0.7–1.5 and O/C ratios between 0.1–0.67 were operationally defined as carboxyl-rich alicyclic molecules (CRAM-like) [27,28,29]. This class of compounds is considered a key molecular signature of RDOC. To evaluate the relative contribution of RDOC, we calculated the proportion of the summed peak intensity of all CRAM-like formulas to the total summed peak intensity of all assigned formulas (the fluorescence intensity contribution) for each sample [30].

To further elucidate changes in the overall structural characteristics of the RDOC molecular pool, two parameters were calculated sequentially for all molecular formulas identified as CRAM-like: the modified aromaticity index (AI_mod_) [31] and the nominal oxidation state of carbon (NOSC) [32]. AI_mod_ reflects the degree of aromatic ring condensation in a molecule, calculated as AI_mod_ = (1 + C-0.5O-0.5N-0.5H-0.5P)/(C-0.5O-N-S-P). NOSC characterizes the overall redox state of a molecule, calculated as NOSC = 4-(4C + H-3N-2O-2S)/C. The arithmetic mean values of AI_mod_ and NOSC for all CRAM-like molecules within each sample were computed. A systematic comparison of these mean values across different treatment groups allowed for an assessment of the dynamic differences in the average aromaticity level and the average redox state of the RDOC fraction, thereby informing the evaluation of its chemical stability and potential degradability [33].

### 2.6. DNA and RNA Extraction and Sequencing

For each time point (days 0, 2, and 28), water samples were collected from the same microcosm bottle. An adequate volume was taken and then split into two equal portions. One portion was filtered through a 0.22 μm glass fiber membrane for DNA extraction, and the other portion was filtered through a separate 0.22 μm glass fiber membrane for RNA extraction. Thus, DNA and RNA were isolated from the same water sample but from different filter membranes, avoiding cross-interference between the two nucleic acid pools. To obtain total DNA from environmental water samples, microbial cells were first collected by filtering the water through a 0.22 μm glass fiber membrane (Delv New Material Co., Ltd., Hangzhou, China). Total genomic DNA was then extracted from the membrane using a commercial DNA extraction kit (Omega Bio-Tek, Norcross, GA, USA) according to the manufacturer’s instructions. The integrity of the extracted DNA was assessed by 1% agarose gel electrophoresis, and its concentration and purity were measured using a NanoDrop 2000 spectrophotometer (Thermo Scientific, Waltham, MA, USA). For RNA extraction, total RNA was extracted from water samples using a commercial RNA extraction kit (Omega Bio-Tek, Norcross, GA, USA) following the manufacturer’s protocol. RNA integrity was assessed by 1% agarose gel electrophoresis, and its concentration and purity were measured using a NanoDrop 2000 spectrophotometer. Ribosomal RNA was removed using a RiboCop rRNA Depletion Kit to enrich messenger RNA.

For 16S rRNA gene amplicon sequencing, the V3–V4 hypervariable region was amplified using primers 338F and 806R [34]. PCR conditions followed Lu et al. [35]: 95 °C for 3 min; 28 cycles of 95 °C for 30 s, 55 °C for 30 s, and 72 °C for 45 s; and a final extension at 72 °C for 5 min. Amplification was performed with TransStart Fastpfu DNA Polymerase on an ABI GeneAmp^®^ 9700 PCR system, with three technical replicates per sample. The purified amplicons were sequenced on an Illumina platform (Illumina, San Diego, CA, USA) using a paired-end (PE250/PE300) strategy with dual-index library construction. For metagenomic sequencing, qualified DNA was fragmented to 350 bp using a Covaris M220 and paired-end libraries were constructed with the TruSeq™ DNA PCR-Free Library Prep Kit (Illumina, USA). Sequencing was performed on the Illumina NovaSeq 6000 platform (PE150) [36]. For metatranscriptomic sequencing, strand-specific libraries were prepared using the TruSeq™ Stranded Total RNA Library Prep Kit (Illumina, USA) with dUTP incorporation. Sequencing was performed on the Illumina NovaSeq 6000 platform (PE150) [37]. All sequencing services were outsourced to Majorbio Bio-Pharm Technology Co., Ltd. (Shanghai, China).

### 2.7. 16S rRNA Gene Amplicon Data Analysis

After sequencing, raw data were first quality-controlled using Fastp (v0.23.4) [38] to remove low-quality bases and reads containing Ns (ambiguous bases). Paired-end reads were then merged using FLASH (v1.2.11) [39], with a minimum overlap of 10 bp and a maximum mismatch rate of 0.2. Following the QIIME2 (v2022.2) [40] pipeline, merged sequences were denoised with the DADA2 plugin [41] to obtain high-resolution amplicon sequence variants (ASVs). Sequences annotated as chloroplast or mitochondrial were removed. To ensure consistent analytical depth, all samples were rarefied to 20,000 sequences. Taxonomic annotation of ASVs was performed using the Naive Bayes classifier with the SILVA (v138) [42] 16S rRNA database as reference, setting a confidence threshold of 0.7 [43]. Alpha diversity indices, including Chao1 and Shannon, were calculated using mothur (v1.30) [44]. Within the R environment (v3.3.1) [45], the vegan package was used to perform principal coordinate analysis (PCoA) and non-metric multidimensional scaling (NMDS) to evaluate between-group differences in community structure (beta-diversity). Statistical significance was tested with PERMANOVA based on 999 permutations [46,47]. Bar charts depicting relative abundance were generated using R packages (ggplot2) [48], displaying only the top 20 taxa at the family and genus levels, with the remainder grouped as “Others”. Community heatmaps were simultaneously constructed at the same taxonomic levels to reveal similarities among samples and the distribution patterns of key taxa. This workflow was implemented by integrating Python (v2.7) and R scripts [49]: the ASV table was first merged according to the five sample groups, the average abundance of each taxon per group was calculated, and the top 20 most abundant taxa were selected for subsequent analysis.

### 2.8. Metagenomic Data Analysis

The raw sequencing data were first quality-controlled using fastp (v0.23.2) [38], which included adapter trimming, removal of low-quality bases (quality score < Q20), and filtering of reads shorter than 50 bp. High-quality sequences after QC were then de novo assembled using MEGAHIT (v1.2.9) [50,51]. With default parameters to generate contig sequences. Open reading frame (ORF) prediction was performed on the assembled contigs using Prodigal software (v2.6.3) [52], which also translated the predicted genes into amino acid sequences. Only ORFs with lengths ≥ 100 bp were retained for subsequent analysis. All predicted gene sequences from all samples were clustered using the CD-HIT tool (v4.6.1) [53] with parameters set at 90% identity and 90% coverage [54]. The longest sequence from each cluster was chosen as the representative to construct a non-redundant gene set. Finally, high-quality reads from each sample were aligned to this non-redundant gene set using SOAPaligner (soap2.21release) [55] with an identity threshold of 95%, and the abundance of each gene in each sample was quantified. For functional annotation, the predicted gene sequences were aligned against the COG and KEGG databases using Diamond (v2.0.13) [56] in blastp mode with an E-value threshold of ≤1 × 10^−5^. The functional annotation and classification methods referenced previously reported approaches for microbial genome analysis [57]. Functional profiles were constructed by summarizing gene abundances across samples at the COG functional category and KEGG pathway levels. To visually compare functional composition differences among treatment groups, the normalized functional abundance matrix was visualized as a heatmap using the ChiPlot (https://www.chiplot.online/) [58].

The assembled contigs were further binned to metagenome-assembled genomes (MAGs) via MetaWRAP with the MaxBin2, metaBAT2 and CONCOCT binning modules [59]. Subsequently, to acquire high-quality MAGs, the bin_refinement module of MetaWRAP was used to integrate the binning results and deduplicated via dRep v3.4.2, with an average nucleotide identity (ANI) of ≥95% [60]. Thresholds were set at ≥50% for completeness and ≤10% for contamination, and an optimized MAG set was generated using a consensus voting strategy [61]. Targeting the key functional genera of interest in this study (*Comamonas*, *Citrobacter*, and *Reyranella*), corresponding MAGs were screened from the medium- and high-quality MAG pool based on taxonomic annotation results generated by GTDB-Tk v2.3.2 [62] The eggNOG-mapper v2.1.9 was used for functional gene classification based on the COG and KEGG databases on the selected MAGs [63]. To quantitatively assess the relative abundance of key MAGs in the samples, high-quality sequencing reads from each sample after quality control were mapped back to the reference genome set composed of all MAG sequences using Bowtie2 (v2.4.5) [64].

### 2.9. Metatranscriptomics Data Analysis

Raw RNA-seq reads were quality-controlled using fastp (v0.23.2) [38] to remove adapter sequences, filter low-quality bases (quality score below Q20), and discard reads shorter than 50 bp. Subsequently, SortMeRNA (v4.3.6) was used to align against the SILVA database to remove rRNA sequences, obtaining high-quality non-rRNA reads [65]. The rRNA-depleted high-quality reads were then assembled de novo using Trinity (v2.15.1) with default parameters to obtain transcript sequences [65]. Open reading frames (ORFs) were predicted from the assembled transcripts using Prodigal (v2.6.3), and gene sequences with a length of no less than 100 bp were retained [52]. The predicted gene sequences from all samples were clustered using CD-HIT (v4.6.1) [53] with parameters set to 90% identity and 90% coverage. The longest sequence from each cluster was selected as the representative to construct a non-redundant gene set [54]. Finally, high-quality reads from each sample were aligned to the non-redundant gene set using SOAPaligner (soap2.21release) [55]. Gene expression abundance was quantified and normalized as transcripts per million (TPM) [66].

The predicted genes were aligned against the COG and KEGG databases using Diamond (v2.0.13) [56] software in blastp mode, with an E-value threshold set at ≤1 × 10^−5^, to acquire functional classification and metabolic pathway annotation information for the genes. Based on the COG category and KEGG pathway abundance data, ChiPlot (https://www.chiplot.online/ was used to visually display functional expression differences among different treatment groups [58]. Subsequently, differential expression analysis was performed using DESeq2 according to sample groupings, and the results were visualized by a differential expression scatter plot (MA plot). To interpret the biological significance of the differentially expressed genes, KEGG pathway enrichment analysis was conducted separately for significantly upregulated and downregulated genes [67]. Building on this, to uncover the associations between the microbial community and key metabolic functions, the most significantly differentiated KEGG pathways among groups were selected from the enrichment analysis results [68]. The specific workflow was as follows: First, based on the differential expression results, KEGG Level 3 pathway enrichment was performed for all significantly differentially expressed genes. A dual-filtering criterion was then applied to identify key pathways: (1) the adjusted *p*-value (*p*-adjust) from the enrichment analysis must be <0.05; and (2) the pathway must belong to a core Level-2 pathway significantly responsive to wastewater input (e.g., Cell Motility, Signal Transduction, Membrane Transport) or fall directly under the “Metabolism” category. Using the psych package in the R environment, Spearman correlation analysis was conducted between these pathways and the genus-level microbial community composition obtained from 16S rRNA gene sequencing, thereby identifying microbial taxa significantly associated with these key metabolic processes.

## 3. Results

### 3.1. Domestic Wastewater Input Reshapes DOC Degradation Kinetics and Nutrient Transformation Efficiency

**Figure 1:**
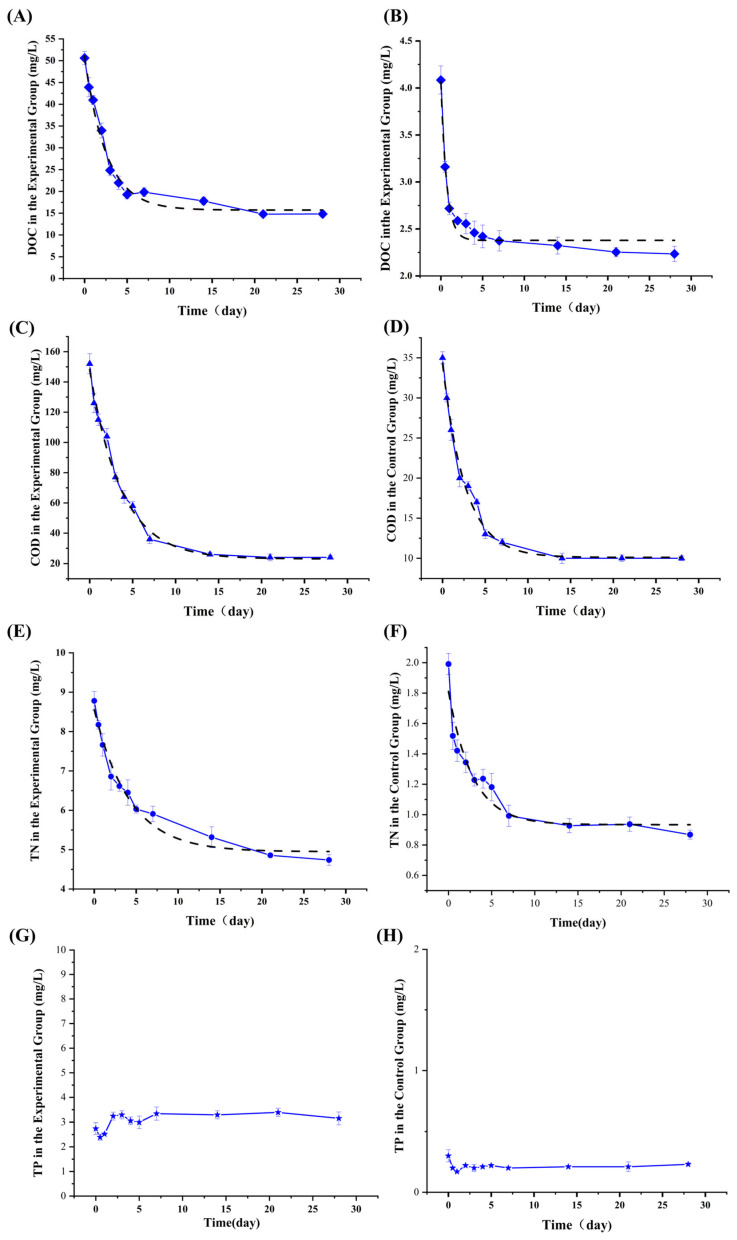
Impacts of domestic wastewater input on physicochemical parameters in microcosm experiments. (**A**) Dynamic changes in dissolved organic carbon (DOC) concentration in the treatment group (Group S) over the 28-day incubation period. The black dotted line represents the exponential fit: y = 35.31·exp(−x/2.55) + 15.72, R^2^ = 0.986. (**B**) Dynamic changes in dissolved organic carbon (DOC) concentration in the control group (Group Q) over the 28-day incubation period. The black dotted line represents the exponential fit: y = 1.69·exp(−x/0.69) + 2.38, R^2^ = 0.970. (**C**) Dynamic changes in chemical oxygen demand (COD) concentration in the treatment group (Group S) over the 28-day incubation period. The black dotted line represents the exponential fit: y = 125.15·exp(−x/3.66) + 23.00, R^2^ = 0.991. (**D**) Dynamic changes in chemical oxygen demand (COD) concentration in the control group (Group Q) over the 28-day incubation period. The black dotted line represents the exponential fit: y = 24.20·exp(−x/2.65) + 10.10, R^2^ = 0.990. (**E**) Dynamic changes in total nitrogen (TN) concentration in the treatment group (Group S) over the 28-day incubation period. The black dotted line represents the exponential fit: y = 3.61·exp(−x/4.18) + 4.95, R^2^ = 0.977. (**F**) Dynamic changes in total nitrogen (TN) concentration in the control group (Group Q) over the 28-day incubation period. The black dotted line represents the exponential fit: y = 0.88·exp(−x/2.77) + 0.94, R^2^ = 0.913. (**G**) Dynamic changes in total phosphorus (TP) concentration in the treatment group (Group S) over the 28-day incubation period. (**H**) Dynamic changes in total phosphorus (TP) concentration in the control group (Group Q) over the 28-day incubation period.

Key environmental parameters were compared between the control group (Group Q, unamended Qiantang River water) and the treatment group (Group S, amended with simulated domestic wastewater) during a 28-day incubation (Figure 1). The initial dissolved organic carbon (DOC) concentration was significantly higher in Group S than in Group Q (Figure 1A,B). DOC in Group S subsequently declined in a decelerating manner, with exponential fitting yielding a decay constant of 2.547 and a half-life of 1.765 days, culminating in a 70% reduction by day 28 (Figure 1A). In contrast, DOC in Group Q started at a lower concentration and declined more gradually, with a decay constant of 0.685 and a half-life of 0.475 days, resulting in a final removal rate of 44%. Temporal changes in chemical oxygen demand (COD) revealed that the initial COD value was substantially higher in Group S than in Group Q (Figure 1C,D). Over time, COD in Group S exhibited a biphasic decline: initially slow, followed by accelerated removal, resulting in an overall reduction of 87%. The fitted curve showed a degradation trajectory with initially slow but ultimately extensive removal. In Group Q, COD decreased steadily throughout the incubation, with a final removal rate of 72%. Total nitrogen (TN) dynamics showed that the initial TN concentration was considerably higher in Group S than in Group Q (Figure 1E,F). TN removal in Group S had a decay constant of 4.18 and a removal rate of 40% by day 28. In contrast, TN decreased more rapidly in Group Q, with a decay constant of 2.77 and a removal rate of 60%. Total phosphorus (TP) concentrations remained relatively stable across both groups throughout the 28-day incubation, with no discernible trends (Figure 1G,H). This stability indicates that phosphorus dynamics were not significantly influenced by sewage input or shifts in C/N ratio under the conditions of this experimental system.

### 3.2. Impact of Wastewater Input on the Composition and Stability of the RDOC Pool

**Figure 2:**
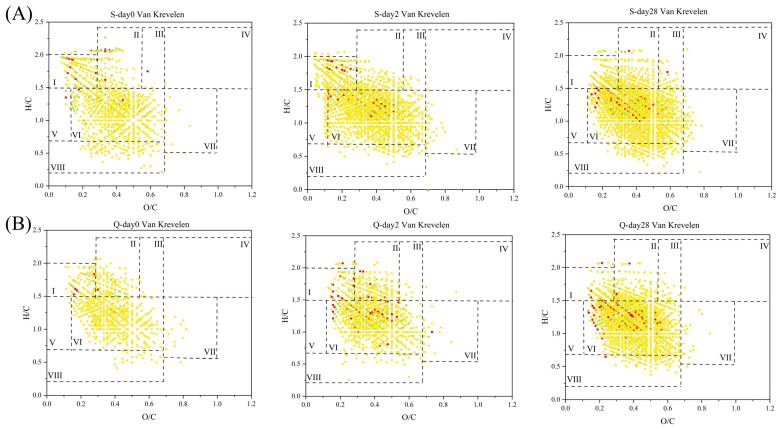
Dynamic evolution of dissolved organic matter molecular composition (Van Krevelen diagrams) and structural characteristics of CRAM-like substances under different C/N conditions. (**A**) 0 and 28 in the experimental group (Group S, C/N ≈ 6). (**B**) Molecular composition distribution (Van Krevelen diagram) of dissolved organic matter on days 0, 2, and 28 in the control group (Group Q, C/N ≈ 3). In the diagrams, each point represents a unique molecular formula identified by FT-ICR MS, with the horizontal coordinate representing the oxygen-to-carbon atomic ratio (O/C) and the vertical coordinate representing the hydrogen-to-carbon atomic ratio (H/C). The color gradient of the points, from red through yellow to green, represents a decreasing trend in normalized mass spectral peak intensity, visually reflecting the relative abundance of each molecule from high to low. According to the classical molecular region classification, eight major categories are annotated in the diagrams: I: Lipids; II: Proteins; III: Amino sugars; IV: Carbohydrates; V: Unsaturated hydrocarbons; VI: CRAM-like substances (carboxyl-rich alicyclic molecules); VII: Tannins; VIII: Condensed aromatics.

**Figure 3:**
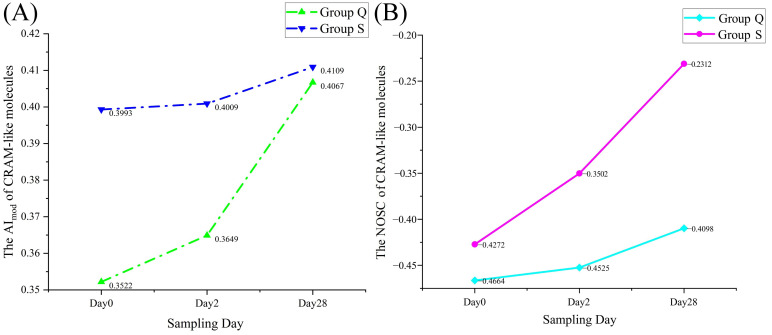
Structural characteristics of CRAM-like compounds under different C/N ratios (**A**) Changes in the average aromaticity index (AI_mod_) of CRAM-like molecules. (**B**) Changes in the average nominal oxidation state of carbon (NOSC) of CRAM-like molecules.

To evaluate the RDOC pool, carboxyl-rich alicyclic molecule-like compounds (CRAM-like) were used as molecular markers, combined with FT-ICR MS analysis and molecular structural indices including the modified aromaticity index (AI_mod_) and the nominal oxidation state of carbon (NOSC) (Figure S1, Figure 2 and Figure 3).

The addition of sewage significantly altered the composition and structural characteristics of the RDOC pool (Figure S1, Figure 2 and Figure 3). The fluorescence intensity contribution of CRAM-like molecules on day 28 was 65.57% in Group S and 69.12% in Group Q. Regarding molecular structural indices, during the incubation period (days 0, 2, and 28), the average AI~mod~ of CRAM-like substances in Group S was 0.3993, 0.4009, and 0.4109, respectively, representing an increase of 0.0116 from day 0 to day 28. In contrast, Group Q showed an increase from 0.3522 to 0.4067, an increase of 0.0545. The average NOSC in Group S increased from −0.4272 to −0.2312 (Δ = +0.1960), while in Group Q it increased from −0.4664 to −0.4098 (Δ = +0.0566).

Based on Table 1 and Figure 2 and Figure 3, on day 0, compared to Group Q, Group S showed a higher number of molecules in the lipid (I), protein (II), CRAM-like (VI), and condensed aromatic (VIII) categories. A small number of highly fluorescent molecules appeared in the CRAM-like region in Group S. By day 2, the total number of molecules increased in both groups; however, Group S still retained more lipid and CRAM-like molecules than Group Q. Additionally, while the number of CRAM-like molecules increased in Group S, the number of strongly fluorescent molecules within this category was lower than in Group Q.Over the 28-day incubation, both groups exhibited a slight increase in the number of molecules within components II (protein-like) and III (amino sugar-like). In Group S, the number of lipid-like molecules (component I) showed a clear gradual decrease from day 2 to day 28.

### 3.3. Changes in Microbial Communities Based on 16S rRNA

**Figure 4:**
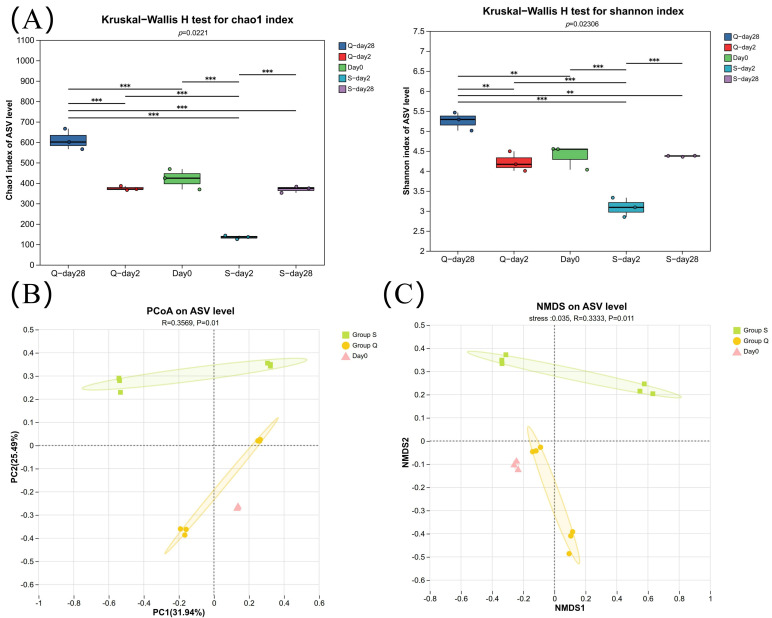
Alpha and beta diversity of microbial communities in response to domestic wastewater input. (**A**) Alpha diversity analysis. Box plots based on the Chao1 and Shannon indices, showing the comparison of microbial community alpha diversity between the control group (Q) and the wastewater-amended group (S) on days 2 and 28. The day 0 sample (labeled as Day 0) was common to both groups as the initial water was identical. (**B**,**C**) Beta diversity analysis. Principal coordinate analysis (PCoA, **left**) and non-metric multidimensional scaling (NMDS, **right**) based on Bray–Curtis distances, revealing differences in community structure between different treatment groups. The first two axes of the PCoA explain 58% of the variation, and the NMDS stress value is 0.035. This figure shows the significant differences between the two selected groups of samples. For pairs with significant differences, markers are assigned as follows: 0.05, ** for 0.001 < *p* ≤ 0.01, and *** for *p* ≤ 0.001.

Based on 16S rRNA gene amplicon sequencing, a total of 2323 ASVs were obtained, which were taxonomically assigned to 35 phyla, 257 families, and 469 genera. Alpha diversity analysis showed that the Chao1 and Shannon indices of Group S were significantly lower than those of Group Q at day 2 and day 28 (*p* < 0.05) (Figure 4A). Beta diversity analysis was performed using principal coordinate analysis (PCoA) based on Bray-Curtis distance, which explained 58% of the variation along the first two axes, and non-metric multidimensional scaling (NMDS) yielded a stress value of 0.035 (<0.05) (Figure 4B,C). Samples from Groups Q and S were clearly separated in both PCoA and NMDS results.

**Figure 5:**
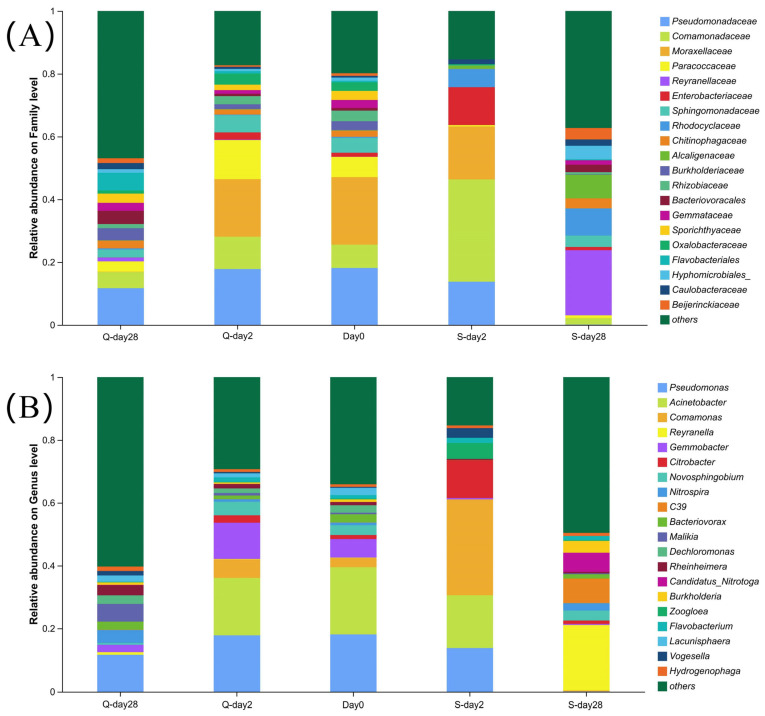
Microbial community composition at family and genus levels under sewage input condition (**A**) Community composition at the family level. Bar charts display the relative abundance of microbial communities at the taxonomic family level for groups Q and S at different incubation time points (0, 2, and 28 days). Only the top 20 most abundant families are shown, with the remainder grouped as “Others”. (**B**) Community composition at the key genus level. Bar charts display the relative abundance of microbial communities at the taxonomic genus level for groups Q and S at different incubation time points (0, 2, and 28 days). Only the top 20 most abundant genera are shown, with the remainder grouped as “Others”.

At the family and genus levels, community composition analysis revealed clear multi-stage changes in the microbial community (Figure 5A,B and Figure S2). At the initial stage (Day 0), the microbial community was dominated by *Moraxellaceae* (21.50%), *Pseudomonadaceae* (18.11%), *Comamonadaceae* (7.47%), and *Paracoccaceae* (6.43%). The corresponding dominant genera were *Acinetobacter* (21.45%), *Pseudomonas* (18.07%), *Comamonas* (2.97%), and *Gemmobacter* (5.88%).

During the short-term response phase (Day 2), Group S exhibited pronounced shifts: *Comamonadaceae* increased sharply to 32.60%, followed by the emergence of *Enterobacteriaceae* (12.08%), while *Paracoccaceae* declined to a negligible level. At the genus level, *Comamonas* became the dominant taxon (30.48%), and *Citrobacter* increased sharply to 12.08%. In contrast, the community structure of Group Q remained largely stable, with only a moderate increase in *Paracoccaceae* (12.44%) and its representative genus *Gemmobacter* (11.58%).

By the long-term adaptation phase (Day 28), the community in Group S underwent substantial restructuring: *Reyranellaceae* (20.70%) and *Rhodocyclaceae* (8.60%) became the dominant families, replacing the previously abundant *Comamonadaceae* and Enterobacteriaceae. At the genus level, *Reyranella* (20.70%) emerged as the predominant taxon, while the relative abundances of *Comamonas* and *Citrobacter* markedly declined. In contrast, Group Q maintained a stable community composition, remaining dominated by *Pseudomonadaceae* (11.70%) and its representative genus *Pseudomonas* (11.60%).

### 3.4. Multi-Omics Reveals the “Stimulus-Balance” Response Pattern of Functional Genes and Metabolic Pathways

**Figure 6:**
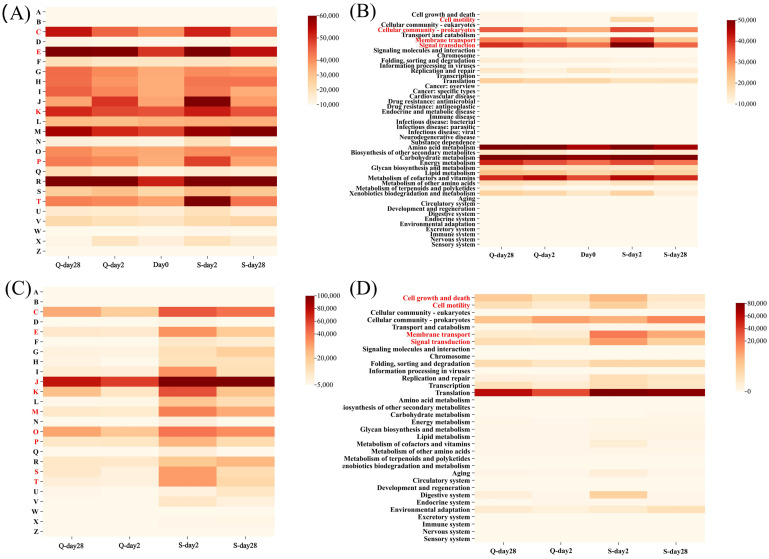
Metagenomic and metatranscriptomic analyses reveal a “stimulus–decline” response pattern of microbial function to wastewater input. (**A**) Heatmap of metagenomic COG functional annotation. Shows the relative gene abundances of COG functional categories at the metagenomic level for the experimental (S) and control (Q) groups on days 0, 2, and 28. Key differentially abundant COG categories are highlighted in red. (**B**) Heatmap of metagenomic KEGG Level-2 pathway annotation. Displays the relative gene abundances of KEGG Level-2 pathways at the metagenomic level. Key differentially abundant KEGG Level-2 pathways are highlighted in red. (**C**) Heatmap of metatranscriptomic COG functional expression. Presents the relative expression abundances of the same core COG functional categories as in (**A**) at the transcriptional level. Key differentially expressed COG categories are highlighted in red. (**D**) Heatmap of metatranscriptomic KEGG Level-2 pathway expression. Illustrates the relative expression patterns of KEGG Level-2 pathways at the metatranscriptomic level. Key differentially expressed KEGG Level-2 pathways are highlighted in red.

Metagenomic and metatranscriptomic analyses were performed to assess functional patterns. In metagenomic analysis, COG functional annotation showed that in Group Q, the abundance of genes associated with energy production and conversion (C), amino acid transport and metabolism (E), transcription (K), inorganic ion transport and metabolism (P), and signal transduction (T) increased steadily throughout the incubation (Figure 6A). In Group S, these functional categories exhibited a pronounced increase on day 2, followed by a decline to near-initial levels by day 28. KEGG Level 2 pathway analysis (Figure 6B) showed that in Group S, pathways related to signal transduction and environmental responsiveness—including Cell Motility, Cellular Community—prokaryotes, Signal Transduction, and Membrane Transport—showed an early surge in gene abundance on day 2, followed by a subsequent decrease. Their corresponding Level 3 pathways, such as Flagellar assembly, Quorum sensing, Two-component system, and ABC transporters, displayed the same trend. In Group Q, these pathways increased continuously over time.

In metatranscriptomic analysis, the expression profiles of COG categories mirrored the metagenomic patterns (Figure 6C). In Group S, core functional categories (C, E, K, P, and T), along with Translation, ribosomal structure and biogenesis (J), Cell wall/membrane/envelope biogenesis (M), and Posttranslational modification, protein turnover, chaperones (O), were upregulated on day 2 but downregulated by day 28. KEGG expression analysis (Figure 6D) showed that Level 2 pathways including Cell Motility, Signal Transduction, Membrane Transport, and Cell Growth and Death all showed an initial increase followed by a decline in Group S, with corresponding Level 3 pathways (e.g., Flagellar assembly, Two-component system, ABC transporters, Cell cycle-Caulobacter) exhibiting the same pattern.

Further analysis focused on the core COG functions (Table S1) and KO units (Table S2) that showed higher abundance on day 2 than on day 28 in Group S. Among the COG annotations belonging to categories C, E, K, P, and T, the following genes had higher abundance on day 2: COG1028 (NAD(P)-dependent dehydrogenase), COG0683 (ABC-type branched-chain amino acid transport system), COG2165 (type II secretory pathway, pseudopilin PulG), COG0642 (signal transduction histidine kinase), COG4771 (outer membrane receptor for ferric siderophore), COG1595 (RNA polymerase sigma subunit), COG0840 (methyl-accepting chemotaxis protein), and COG2199 (diguanlyate cyclase). In the KEGG database, functional genes associated with transmembrane transport and signal sensing, including K03406 (methyl-accepting chemotaxis protein), K02014 (ferric complex outer membrane receptor protein), K01999 (branched-chain amino acid transport system substrate-binding protein), K07795 (tricarboxylate transport membrane protein), K12132 (serine/threonine protein kinase), K02406 (flagellin), and K21449 (trimeric autotransporter adhesin), also showed higher abundance on day 2.

In metatranscriptomic data, the core COG functions (Table S3) and KO units (Table S4) that showed higher expression on day 2 included COG0642 (histidine kinase), COG4771 (ferric siderophore receptor), K03406 (chemotaxis receptor protein), and K02406 (flagellin). These genes were upregulated at S-day2 and downregulated by S-day28. Cross-referencing of the four datasets identified a core set of functional genes that consistently showed higher abundance/expression on day 2 than on day 28 in Group S. This set includes genes related to transmembrane signal recognition and transduction (COG0642, K03406, K11527, K12132), iron acquisition and transport (COG4771, K02014), motility and adhesion (K02406, K21449, COG2165), and stress response to exogenous substances.

### 3.5. Association Between Key Taxa and Metabolic Functions Reveals the Driving Mechanism of the Microbial Carbon Pump

#### 3.5.1. Early-Stage Xenobiotic Degradation

Differential gene expression analysis was conducted between the experimental and control groups at key time points (S-day2 vs. Q-day2, S-day28 vs. Q-day28) (Figure S3). In Group S on day 2, significantly enriched pathways (*p*-adjust < 0.05) included Dioxin degradation, Polycyclic aromatic hydrocarbon degradation, Caprolactam degradation, and Atrazine degradation, all belonging to the KEGG second-level category of Xenobiotics biodegradation and metabolism (Figure S3A). Key responsive genes within these pathways included K18366 and K02554 (Dioxin degradation pathway), and K01428 and K23359 (Atrazine degradation pathway).

#### 3.5.2. Late-Stage Endogenous Synthesis

In Group S on day 28, enriched pathways shifted to Purine metabolism, Valine, leucine and isoleucine biosynthesis, Lysine biosynthesis, and Pyruvate metabolism, belonging to the KEGG second-level categories of Nucleotide metabolism, Amino acid metabolism, and Carbohydrate metabolism (Figure S3B). Upregulated genes at this stage included K01652 and K01649 (Valine, leucine and isoleucine biosynthesis) and K00133 and K01714 (Lysine biosynthesis pathway).

In Group Q on day 2, the main enriched pathways were Fatty acid degradation and Nonribosomal peptide structures, belonging to the KEGG second-level categories of Lipid metabolism and Metabolism of terpenoids and polyketides (Figure S3C). On day 28, the enriched pathways in Group Q shifted to Arginine biosynthesis, Biosynthesis of unsaturated fatty acids, Biosynthesis of type II polyketide products, and Degradation of aromatic compounds, affiliated with the KEGG second-level categories of Amino acid metabolism, Lipid metabolism, and Metabolism of terpenoids and polyketides (Figure S3D).

#### 3.5.3. Spearman Taxon-Functional Coupling

**Figure 7:**
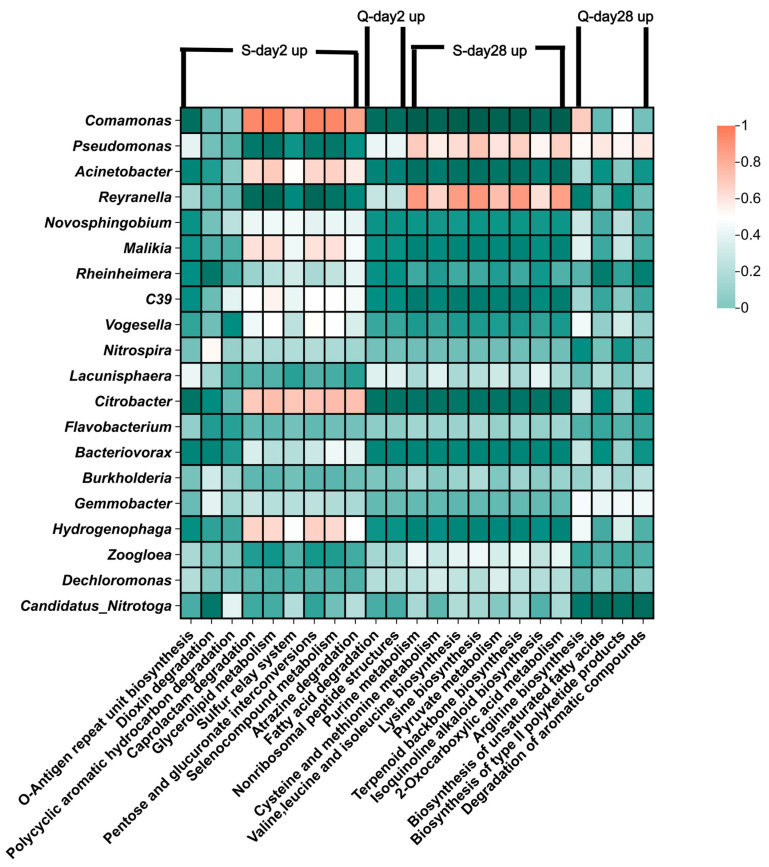
Correlation network diagram between genus-level microbial community and differential metabolic pathways.

Subsequently, based on the KEGG pathway enrichment results and genus-level species composition data, we conducted Spearman correlation analysis to explore the key microbial taxa in the aforementioned functional succession (Figure 7). The results showed significant differentiation in the core microbial communities and their associated metabolic functions under different treatment conditions, forming a complete response chain from short-term stress to long-term adaptation, which directly regulated the production efficiency and chemical properties of RDOC. During the short-term stress period (S-day2), *Comamonas* and *Citrobacter* emerged as the dominant genera. Both showed strong positive correlations (r > 0.7, *p* < 0.05) with pathways such as Caprolactam degradation, Glycerolipid metabolism, Selenocompound metabolism, and Atrazine degradation. This indicates that in the initial phase of wastewater input, these degradative microbial groups acted as a “metabolic engine” for rapidly transforming exogenous complex pollutants, accelerating the mineralization of readily degradable organic matter. However, this rapid decomposition-oriented metabolic strategy likely inhibited the formation of complex and stable RDOC components through microbial synthesis and modification, mechanistically explaining the lower aromaticity (AI_mod_) of RDOC molecules in the short term. By the long-term adaptation period (S-day28), the dominant microbiota had shifted, with *Reyranella* becoming the predominant genus. Its metabolic focus shifted from exogenous pollutant degradation to endogenous basic synthesis, associating strongly with pathways like Purine metabolism, Valine, leucine and isoleucine biosynthesis, Lysine biosynthesis. This reflects the microbial community’s transition under altered C/N stoichiometry from a “stress response” to an “adaptive maintenance” strategy centered on nitrogen recycling and cellular maintenance. The products of *Reyranella*-dominated anabolic metabolism tend to be structures such as nitrogen-containing heterocycles and polypeptide fragments, characterized by low aromatic condensation and high oxidation levels. Consequently, this leads to an overall RDOC profile with lower average AI_mod_, higher NOSC, and reduced stability, indicating decreased quality. In contrast, in the control group (Group Q) without wastewater input, no single dominant genus showed a strong positive correlation (r > 0.7, *p* < 0.05) with specific pathways. Its metabolic functions were cooperatively performed by various microorganisms (e.g., *Pseudomonas*), presenting a community feature of balanced functional distribution and a stable metabolic network. This cooperative and sustained metabolic pattern facilitates the slow transformation and molecular polymerization of organic matter, forming RDOC components with higher aromaticity and more complex structures, thereby supporting a higher carbon sequestration potential.

### 3.6. Genomic Evidence from Metagenome-Assembled Genomes (MAGs)

**Figure 8:**
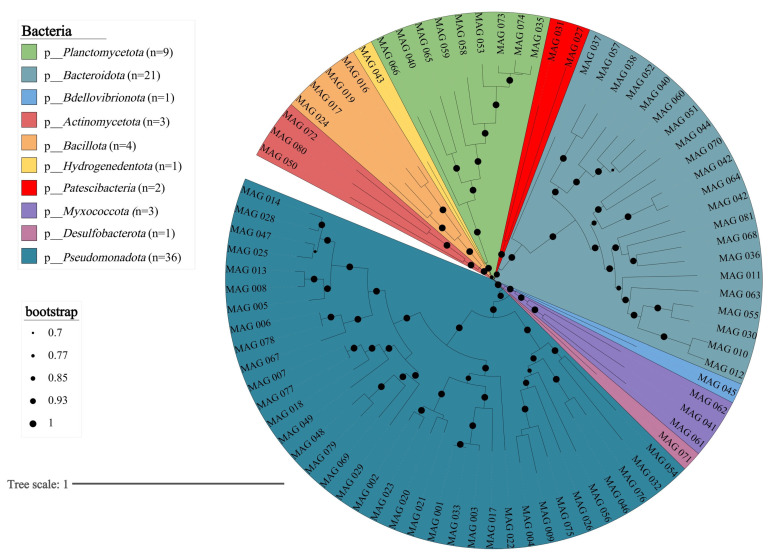
Phylogenetic tree of 81 metagenome-assembled genomes (MAGs) based on GTDB-Tk. Bootstrap support values (≥0.7) are indicated at nodes.

Further analysis was conducted on the metagenome-assembled genomes (MAGs) of the key microbial groups (Figure 8). At S-day2, a total of 31 MAGs were obtained, including 11 high-quality and 20 medium-quality MAGs. These MAGs were taxonomically assigned to four phyla: *Pseudomonadota*, *Bacteroidota*, *Bacillota* and *Patescibacteria*. Among them, MAGs from four key groups were identified: MAG001 (assigned to *Citrobacter freundii*, a relative abundance of 11.66%), MAG005 (*Comamonas testosteroni_B*, 23.80%), MAG008 (*Comamonas aquatica*, 4.79%), and MAG013 (*Comamonas terrigena*, 1.51%). At S-day28, a total of 50 MAGs were obtained, including 14 high-quality and 36 medium-quality MAGs. These MAGs were assigned to nine phyla: *Pseudomonadota*, *Bacteroidota*, *Planctomycetota*, *Myxococcota*, *Hydrogenedentota*, *Bdellovibrionota*, *Desulfobacterota* and *Actinomycetota*. From these, MAGs from two key groups, both of medium quality, were identified: MAG046 (*Reyranella aquatilis*, 16.69%) and MAG076 (*Reyranella massiliensis*, 3.81%). These results were largely consistent with the 16S rRNA gene sequencing results (Figure 5B).

**Figure 9:**
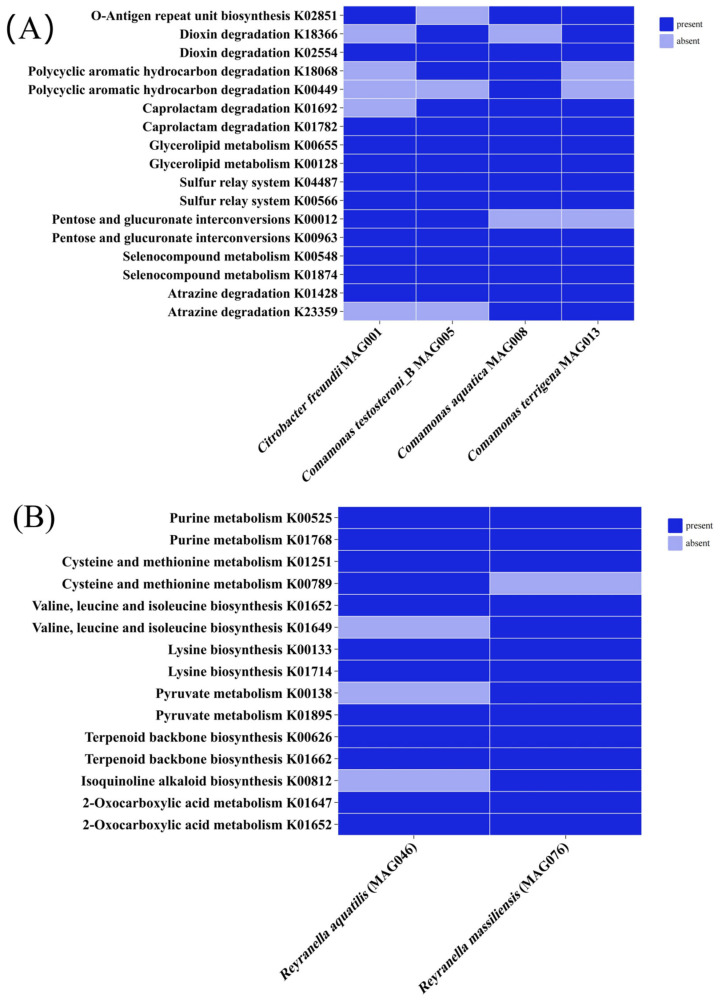
Distribution of key metabolic genes in the genomes of pivotal strains during the stimulus and balance phases. (**A**) Presence of key KO genes related to exogenous pollutant degradation, etc., in the metagenome-assembled genomes of key taxa (*Comamonas*, *Citrobacter*) from Group S on Day 2 (stimulus phase). (**B**) Presence of key KO genes related to endogenous anabolic metabolism, etc., in the metagenome-assembled genomes of key taxa (e.g., *Reyranella*) from Group S on Day 28 (balance phase).

We systematically screened these key MAGs for the presence of key functional orthologs identified by differential expression analysis. The results corroborated the earlier species-function correlation analyses and provided more refined genomic evidence (Figure 8). During the stress phase on day 2 (Figure 9A), the four dominant strains (*Citrobacter freundii* MAG001, *Comamonas testosteroni*_B MAG005, *Comamonas aquatica* MAG008, and *Comamonas terrigena* MAG013) mainly carried genes involved in xenobiotic degradation (dioxin, caprolactam, polycyclic aromatic hydrocarbons, atrazine), glycerolipid metabolism, sulfur relay, pentose/glucuronate interconversions, and selenocompound metabolism. Several orthologs were co-present in all four strains: *mhpD* (K02554), *fadB* (K01782), *plsC* (K00655), *ald* (K00128), *iscS* (K04487), *mnmA* (K00566), *galU* (K00963), *metG* (K01874), and *ureC* (K01428). Other orthologs showed more specific distribution patterns: rfe (K02851) was co-encoded by *C*. *freundii* MAG001, *C. aquatica* MAG008, and *C. terrigena* MAG013; aldH (K18366) was present in *C. testosteroni*_B MAG005 and *C. terrigena* MAG013; *ph3* (K18068) was carried jointly by *C. testosteroni*_B MAG005 and *C. aquatica* MAG008; *ugd* (K00012) was shared by *C. freundii* MAG001 and *C. testosteroni*_B MAG005; and *biuH* (K23359) was distributed in *C. aquatica* MAG008 and *C.* *terrigena* MAG013. These distribution patterns further revealed synergistic and complementary metabolic relationships among the strains.

Upon entering the balance phase on day 28 (Figure 9B), metabolic functions were primarily undertaken by two *Reyranella* strains (*Reyranella aquatilis* MAG046 and *Reyranella massiliensis* MAG076). These strains mainly performed anabolic pathways, including purine metabolism, valine/leucine/isoleucine biosynthesis, lysine biosynthesis, pyruvate metabolism, terpenoid backbone biosynthesis, and cysteine/methionine metabolism. The following orthologs were co-encoded by both strains, indicating synergy in core metabolic pathways: *cyaA* (K01768), *cyaA* (K01251), *ilvB* (K01652), *asd* (K00133), *dapA* (K01714), *acs* (K01895), *atoB* (K00626), *dxs* (K01662), and *gltA* (K01647). Functional differentiation was reflected in their unique genes: R. *aquatilis* MAG046 uniquely possessed metK (K00789), while R. *massiliensis* MAG076 specifically encoded *leuA* (K01649), *aldB* (K00138), and *aspC* (K00812). This distribution pattern reveals a metabolic division of labor during the balance phase, achieved through the sharing of core functions and the differentiation of specialized functions between the two strains.

## 4. Discussion

### 4.1. Coupled Microbial Community Succession and Metabolic Pathway Remodeling Drive the Cascade Mechanism of RDOC Quality Decrease

This study reveals that domestic sewage input significantly reduces microbial community diversity by altering the stoichiometric balance of the water body (increased C/N ratio) and the molecular composition of organic matter. It drives the directional succession of microbial taxa from degradation-oriented to synthesis-oriented types, leading to a temporal “stimulation–balance” response pattern in microbial functions. Ultimately, this reshapes the composition and stability of the RDOC pool, manifesting as a feature of decreased quality [8,10]. This cascade mechanism—“environmental change → community succession → functional remodeling → carbon pool alteration”—elucidates the intrinsic logic of anthropogenic disturbance on estuarine carbon cycling from multiple dimensions.

In the early stage of sewage input (S-day2), the water was enriched with relatively simple and highly bioavailable organic molecules (including lipids and proteins introduced by the sewage, as well as some newly formed RDOC precursors), which provided an ideal ecological niche for bacterial groups such as *Comamonas* and *Citrobacter* possessing strong capabilities for degrading exogenous organic compounds [69,70,71]. 16S rRNA gene amplicon sequencing results showed that sewage input led to a significant decline in microbial community diversity (decreased Chao1 and Shannon indices, increased Simpson index), rapid expansion of dominant taxa, and simplification of community structure. In the early stage of wastewater input, the community became dominated by taxa known for xenobiotic degradation (*Comamonas*, *Citrobacter*), whereas the control group exhibited a more stable composition centered on Pseudomonas (Figure 4). This community succession pattern is highly consistent with the significant decrease in alpha diversity and the prominence of dominant taxa observed by Hou et al. throughout the A2/O wastewater treatment process [72], and it corroborates the findings of Li et al., who reported significantly reduced bacterial diversity and evenness at sewage-impacted sites in urban river sediments [73]. Carles et al. also noted that sewage discharge markedly alters the composition and function of microbial communities in downstream water bodies [74].

Accompanying the dramatic shifts in community structure, cross-referencing of metagenomic and metatranscriptomic data identified a core set of functional genes in microorganisms exhibiting a “stimulation-balance” response mode under sewage stress. This set primarily includes functional genes associated with transmembrane signal recognition and transduction (e.g., COG0642, K03406), iron acquisition and transport (e.g., COG4771, K02014), motility and attachment (e.g., K02406, K21449), and responses to exogenous substance stress (Tables S1–S4). Their expression dynamics essentially serve as a direct functional manifestation of the turnover of dominant taxa [75,76]. Bains et al., in their study on the impact of L-norepinephrine on sewage microbial communities, found that although the carbon content in the treatment group was only one-tenth of that in the control group, it achieved higher biomass growth and induced the coordinated upregulation of oxidative stress genes along with significant modulation of central carbon and nitrogen metabolic pathways [77]. Leao et al., in their study of wastewater treatment plant–stabilization pond systems, observed significant succession of microbial communities along the treatment process, with exogenous sewage-borne bacteria struggling to colonize the stable indigenous community, reflecting the screening effect of ecosystem functions on microbial communities [78].

As the incubation progressed, the microbial community underwent a fundamental restructuring of metabolic pathways [79,80]. Ecological stoichiometry theory posits that an imbalance in resource C:N:P ratios drives microorganisms to adjust their life strategies to maintain internal elemental homeostasis [81,82] This selective pressure favors a shift from r-strategist catabolic-dominant taxa to K-strategist anabolic-dominant taxa. In the early phase, *Comamonas* and *Citrobacter*—typical copiotrophic r-strategists—rapidly exploited abundant labile organic carbon, exhibiting high growth rates and catabolic activation. As nitrogen became limiting, K-strategists (e.g., *Reyranella*) with higher substrate affinity and more efficient nitrogen conservation outcompeted the r-strategists. By the long-term adaptation phase (S-day28), the genus *Reyranella* (20.70%) had become the absolutely dominant taxon, while the signals of the previously dominant *Comamonas* and *Citrobacter* were significantly weakened (Figure 4). Consequently, the metabolic focus shifted from exogenous pollutant degradation to endogenous substance synthesis, particularly pathways such as purine metabolism, branched-chain amino acid and lysine biosynthesis (Figure 6B), which fall under nucleotide metabolism, amino acid metabolism, and carbohydrate metabolism. The core of these “nitrogen-saving” anabolic pathways is the efficient assimilation and fixation of limited nitrogen resources into cellular structural components or secretory products. Their metabolic outputs, such as nitrogen-containing heterocycles (purine and pyrimidine derivatives), polypeptides, and protein fragments, contributed to the molecular diversity of the dissolved organic matter pool. However, molecules produced predominantly through biosynthesis typically have higher nitrogen and oxygen content, underdeveloped aromatic ring condensation structures, and consequently a higher overall oxidation state (NOSC) [83]. Therefore, the characteristics of the products from their dominant metabolic pathways dictated the weaker chemical stability of these newly formed RDOC components (“quality decrease”) [84]. The regulatory role of key microbial groups on RDOC characteristics in other studies also corroborates our findings. In a karstic river study, *Sporichthyaceae* and *Novosphingobium* were identified as key microbial groups driving the formation of microbial-derived dissolved organic carbon (MDOC), with their abundance changes directly correlating with RDOC accumulation [85]. In wastewater treatment plant studies, the successional process of the microbial community exhibited a transition from a degradation-type to a synthesis-type, accompanied by a shift in the molecular composition of effluent dissolved organic matter from simple small molecules to complex nitrogen-containing compounds [86]. In soil remediation research, organic materials with a high C/N ratio promoted the enrichment of oligotrophic K-strategists (e.g., *Actinobacteria*, *Acidobacteria*), while a low C/N ratio drove the rapid proliferation of copiotrophic r-strategists (e.g., *Proteobacteria*, *Firmicutes*). This C/N ratio-driven shift in ecological strategy directly determined the chemical properties of microbial metabolic products [87].

### 4.2. Limitations of Study

While the microcosm experiment provided controlled insights into the response mechanisms of microbial communities to wastewater input, its extrapolation to natural estuarine environments warrants caution. First, the 28-day incubation period, although sufficient to capture early successional dynamics, may not fully represent seasonal or interannual adaptation processes in the field. Second, the closed microcosm system lacks continuous water exchange, tidal fluctuations, and sediment interactions that characterize real estuaries. Nevertheless, the observed directional succession from *Comamonas–Citrobacter* to *Reyranella* and the corresponding “stimulation-balance” functional shift are consistent with field observations from other wastewater-impacted rivers [75,76,88,89], supporting the ecological relevance of our findings. Therefore, the microcosm results should be interpreted as a qualitative demonstration of mechanistic linkages rather than quantitative predictions of in situ transformation rates.

Regarding the sampling time points, the selection of days 0, 2, and 28 for multi-omics analysis was based on preliminary BDOC degradation kinetics. As shown in the pre-experiment, DOC concentrations declined most rapidly within the first two days and then plateaued after day 14, with no significant change between day 21 and 28. Day 2 thus represents the peak of the “stimulation” phase, while day 28 captures the established “balance” phase. However, we acknowledge that the absence of an intermediate time point (e.g., day 14) may have missed finer dynamics, such as the exact timing of the metabolic switch from catabolism to anabolism. Future studies should include additional sampling points (e.g., day 14) and extend incubation periods to seasonal or longer scales to better resolve the transition dynamics and assess the long-term stability of the newly formed RDOC.

Furthermore, this study inferred the metabolic potential of key microbial groups (*Comamonas*, *Citrobacter*, *Reyranella*) through correlation analysis and metagenome-assembled genomes, but their functions have not been directly verified through pure culture experiments. Subsequent work could involve isolating these key strains and conducting functional validation experiments under pure culture conditions to further confirm their specific mechanisms in RDOC generation and transformation. The in-depth development of this work will provide a more solid scientific basis for the anthropogenic regulation of estuarine carbon sink functions.

## 5. Implications

### Implications for Estuarine Carbon Sink Management: Emission Reduction Measures Can Help Restore RDOC Stability and Carbon Sequestration Potential

This study, through microcosm simulation experiments combined with multi-omics technologies, systematically revealed the impact mechanism of domestic sewage input on the dissolved organic carbon pool and microbial community in the Qiantang River. The results showed that domestic sewage input increased the organic carbon load in the water and led to a decrease in the fluorescence intensity contribution of RDOC molecules marked by CRAM-like compounds. Furthermore, the average aromaticity index (AI_mod_) of these molecules showed a relatively small increase, while the average nominal oxidation state of carbon (NOSC) showed a larger increase, indicating a reduction in the chemical stability and degradation resistance of RDOC—decreased quality. Concurrently, sewage input led to a decrease in microbial community diversity and drove a directional succession in community structure: In the initial phase, catabolic-dominant taxa such as *Comamonas* and *Citrobacter* dominated, rapidly activating functional genes and pathways related to exogenous pollutant degradation, transmembrane transport, and signal transduction. In the later phase, the community succeeded in an anabolic-dominant group centered on *Reyranella*, with the metabolic focus shifting towards endogenous maintenance pathways such as amino acid synthesis and purine metabolism. This “stimulation-balance” functional response mode was driven by the turnover of key microbial groups and ultimately led to changes in the composition and stability of the RDOC pool. These findings elucidate, from the multi-dimensional perspective of “organic molecular characteristics—community structure—functional genes,” how sewage input, by reshaping the microbial community and its microbial carbon pump operation mode, affects the estuarine carbon cycle.

The above research results provide important implications for estuarine carbon sink management. Firstly, traditional pollution reduction measures (such as upgrading wastewater treatment plants, controlling non-point source pollution in watersheds, and remediating sewage outfalls into rivers) can not only reduce exogenous pollutant inputs but also help restore the natural stoichiometric balance (especially the C/N ratio) of the water body. This can guide the microbial community towards developing a stable, synergistic metabolic network, promote the generation of RDOC components with high aromaticity and complex structures, and enhance the carbon sequestration potential of the estuarine ecosystem. Secondly, the C/N ratio can serve as a “switch” indicator for regulating the operation of the microbial carbon pump. In watershed pollution control planning, in addition to focusing on the reduction of conventional pollutants, attention should be paid to the elemental stoichiometry of materials entering rivers. By optimizing wastewater treatment processes, adjusting agricultural fertilization structures, and other measures, the microbial community can be guided to succeed in a functional direction favorable for carbon sequestration, achieving synergistic benefits of water quality improvement and enhanced carbon sink capacity.

## Figures and Tables

**Table 1:** Dynamic changes in the number of molecules of each fluorescent component (I–VIII) in groups Q and S during incubation.

	Lipids (I)	Proteins (II)	III: Amino Sugars	IV: Carbohydrates	V: Unsaturated Hydrocarbons	VI: CRAM-Like	VII: Tannins	VIII: Condensed Aromatics
S-day28	185	248	129	1	0	1812	43	124
S-day2	325	230	37	1	70	1002	41	89
S-day0	240	207	11	1	10	663	31	108
Q-day0	149	161	11	0	0	392	23	42
Q-day2	206	216	49	2	1	695	38	57
Q-day28	191	246	113	1	6	1585	59	161

## Data Availability

The raw sequencing data (metagenomic and metatranscriptomic) generated in this study have been deposited in the NCBI Sequence Read Archive (SRA) under BioProject accession number PRJNA1403249 (https://www.ncbi.nlm.nih.gov/bioproject/PRJNA1403249 (accessed on 15 January 2026)). The SRA run accession numbers for the samples are SRR36843373–SRR36843389. The metagenome-assembled genomes (MAGs) are available under the same BioProject, with corresponding BioSample accession numbers SAMN54771964–SAMN54772044.

## Supplementary Materials

The following supporting information can be downloaded at: https://www.mdpi.com/article/10.3390/microorganisms14061282/s1, Figure S1: Number and proportion of CRAM-like molecules in the samples. Figure S2: Temporal dynamics of the microbial community at (A) the family and (B) the genus level across different treatment groups. Figure S3: Differential gene expression, functional enrichment, and association analysis with the microbial community. Table S1: Functional genes exhibiting a stimulus-balance pattern influenced by domestic wastewater input and their abundances, based on metagenomic analysis of the COG database. Table S2: Functional genes exhibiting a stimulus-balance pattern influenced by domestic wastewater input and their abundances based on metagenomic analysis of the KEGG database. Table S3: Functional genes exhibiting a stimulus-balance pattern influenced by domestic wastewater input and their expression levels, based on metatranscriptomic analysis of the COG database. Table S4: Functional genes exhibiting a stimulus-balance pattern influenced by domestic wastewater input and their expression levels, based on metatranscriptomic analysis of the KEGG database. Table S5: Differentially expressed key genes influenced by domestic wastewater input and their associated metabolic pathways.

## Abbreviations

Table following abbreviations are used in this manuscript:

RDOC	Recalcitrant Dissolved Organic Carbon
DOC	Dissolved Organic Carbon
CRAM	Carboxyl-Rich Alicyclic Molecules
AI_mod_	modified Aromaticity Index
NOSC	Nominal Oxidation State of Carbon
MCP	Microbial Carbon Pump
FT-ICR MS	Fourier Transform Ion Cyclotron Resonance Mass Spectrometry
NPOC	Non-Purgeable Organic Carbon
ASV	Amplicon Sequence Variant
PCoA	Principal Coordinate Analysis
NMDS	Non-metric Multidimensional Scaling
TPM	Transcripts Per Million
