# Supplementary material for: Multi-Omics Reveals the Impact of Domestic Wastewater Input on the Dissolved Organic Carbon Pool and Microbial Community in the Qiantang River Estuary
Source: Microorganisms. 2026 Jun 5;14(6):1282. doi: 10.3390/microorganisms14061282 (PMC13303571; doi:10.3390/microorganisms14061282)
Supplement: Supplementary file 1 [file microorganisms-14-01282-s001.zip › microorganisms-4303201-supplementary.pdf]

Article

Multi-omics Reveals the Impact of Domestic Wastewater Input on the Dissolved Organic Carbon Pool and Microbial Community in the Qiantang River Estuary.

Yun-Fei Cao[1], Yi-Ru Wang, Pei-Xin Zheng, Xing-Chen Wang, Lin Xu[1,2], Cong Sun[1,2,*]

[1] College of Life Sciences and Medicine, Zhejiang Sci-Tech University, Hangzhou 310018, PR China; 2023220902006@mails.zstu.edu.cn (Y.-F.C.); yr_1414@163.com (Y.-R.W.);2923378901@qq.com(P.-X.Z.); linxu@zstu.edu.cn (L.X.); michael_sc@sina.com (C.S.)

[2] Shaoxing Biomedical Research Institute of Zhejiang Sci-Tech University Co., Ltd., Zhejiang Engineering Research Center for the Development Technology of Medicinal and Edible Homologous Health Food, Shaoxing 312075, PR China; 1065951184@qq.com (X.-C.W.)

* Correspondence:Cong Sun (Email: michael_sc@sina.com)

Figure S1. Number and proportion of CRAM-like molecules in the samples.
(A) Bar chart showing the total number of molecular formulas identified in each sample and the number of those classified as CRAM-like.
(B) Bar chart showing the relative contribution of CRAM-like molecules to the total fluorescence intensity in each sample.
(C)

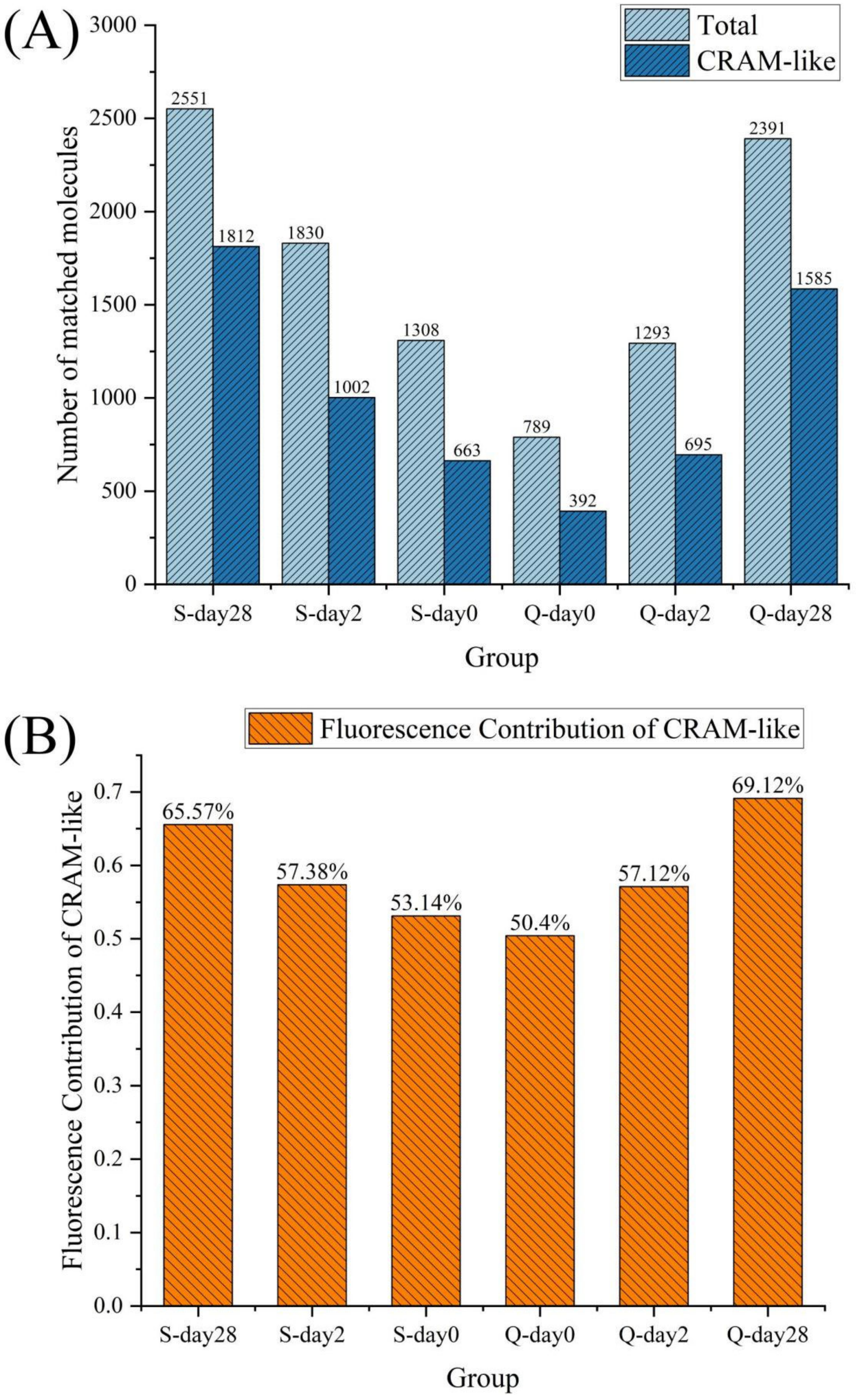

Figure S2. Temporal dynamics of the microbial community at (A) the family and (B) the genus level across different treatment groups.

(A) Community composition at the family level. The heatmap displays the relative abundance of the microbial community at the taxonomic family level for groups Q and S at different incubation time points (0, 2, and 28 days). Only the top 20 most abundant orders are shown, with the remainder grouped as "Others".

(B) Community composition at the key genus level. The heatmap displays the relative abundance of the microbial community at the taxonomic genus level for groups Q and S at different incubation time points (0, 2, and 28 days). Only the top 20 most abundant genera are shown, with the remainder grouped as "Others".

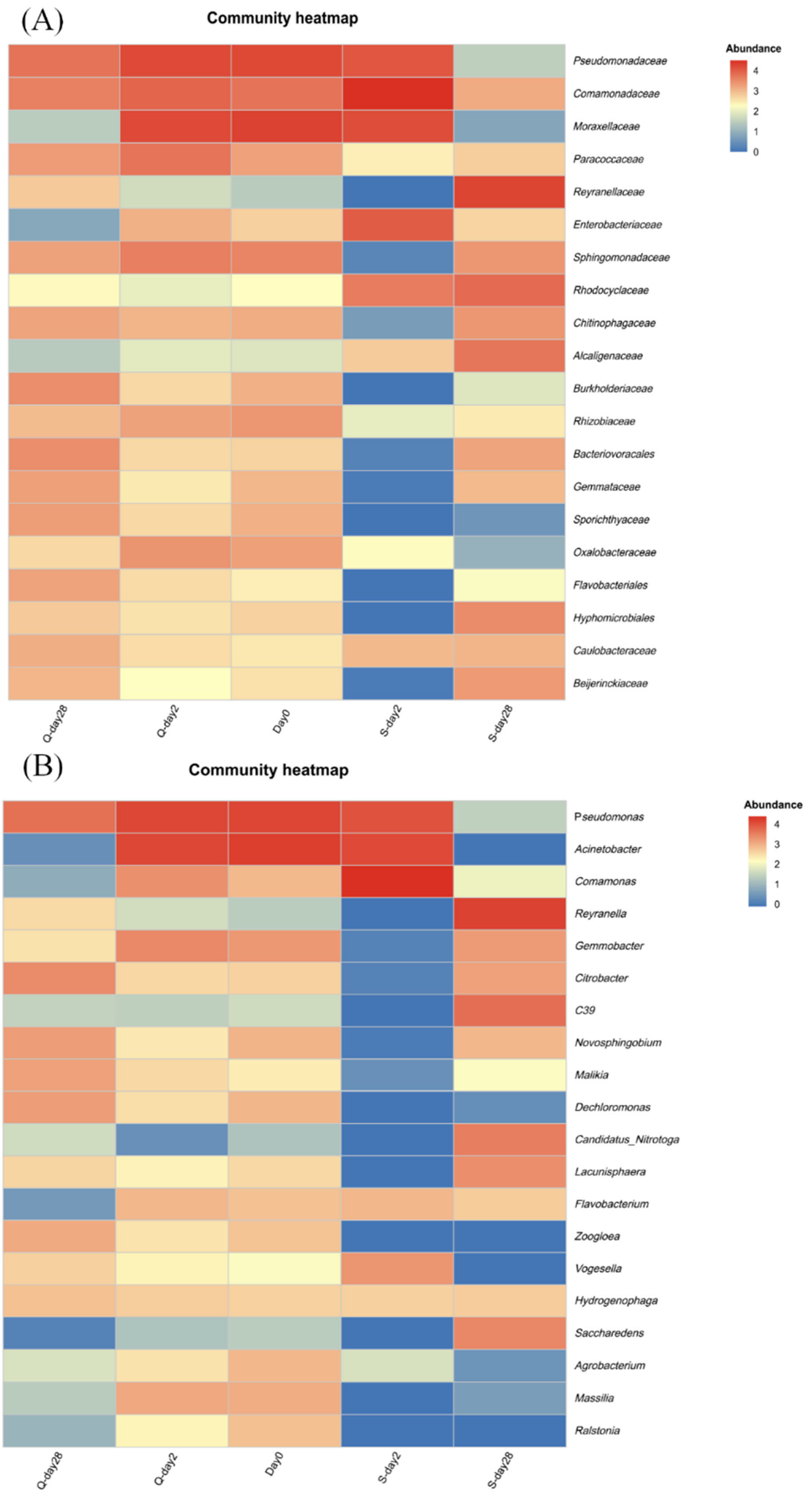

Figure S3. Differential gene expression, functional enrichment, and association analysis with the microbial community. **(A-D)** Bubble charts of KEGG pathway enrichment for differentially expressed genes. Display the KEGG pathway enrichment results for significantly upregulated genes (p-adjust < 0.05) under the following conditions: S-day2 **(A)**, S-day28 **(B)**, Q-day2 **(C)**, and Q-day28 **(D)**. Bubble size represents the number of genes enriched in the pathway, and bubble color indicates the significance level of the enrichment analysis.

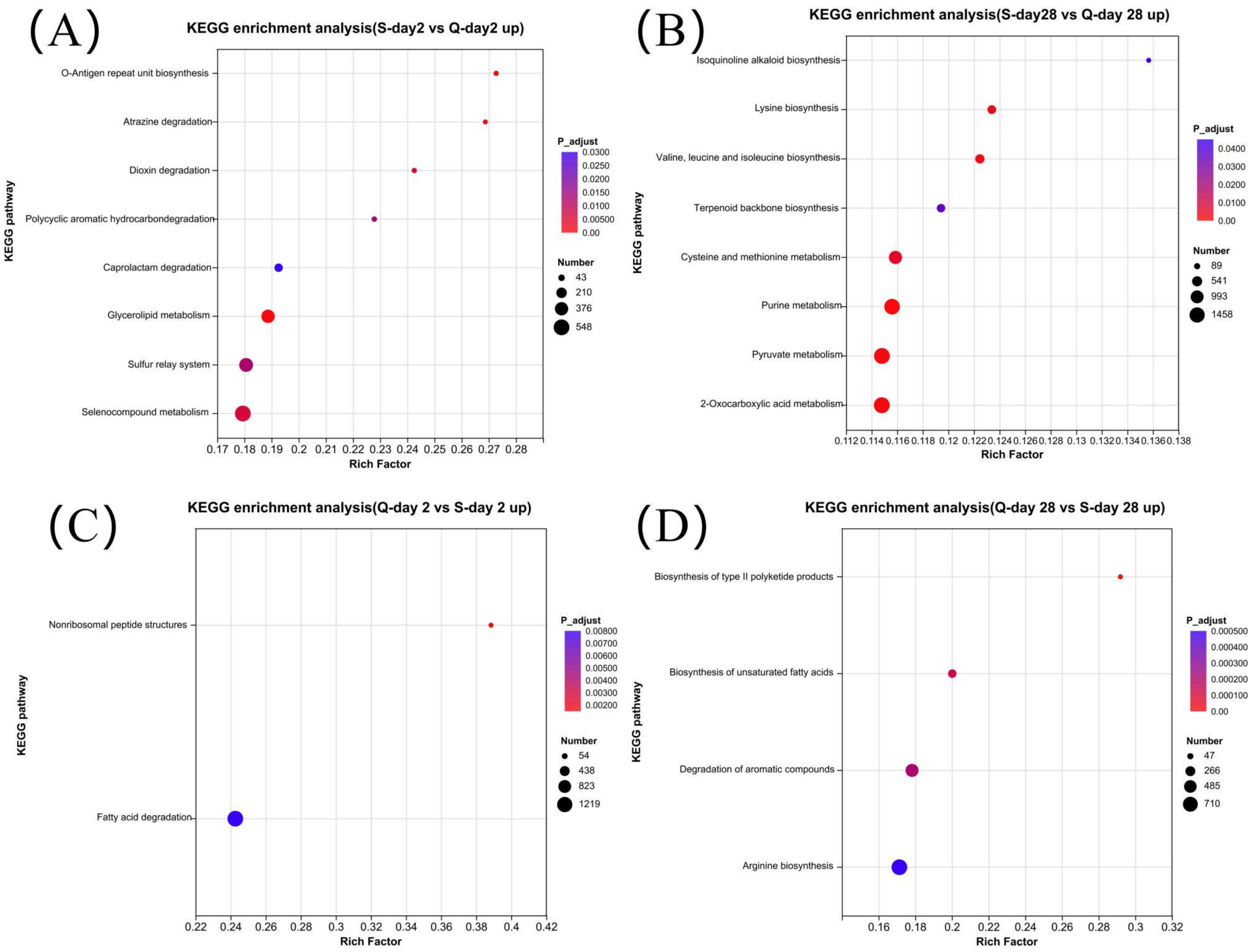

Table S1. Functional genes exhibiting a stimulus-balance pattern influenced by domestic wastewater input and their abundances, based on metagenomic analysis of the COG database.

COG	S-day28 （TPM）	S-day2 （TPM）	Day0 （TPM）	Q-day2 （TPM）	Q-day28 （TPM）	Function	Description
COG2010	1275	3512	1941	2008	2372	C（Energy production and conversion）	Cytochrome c, mono- and diheme variants
COG1249	394	816	468	506	600	C（Energy production and conversion）	Dihydrolipoamide dehydrogenase (E3) component of pyruvate/2-oxoglutarate dehydrogenase complex or glutathione oxidoreductase
COG1053	276	655	299	408	524	C（Energy production and conversion）	Succinate dehydrogenase/fumarate reductase, flavoprotein subunit
COG1028	3745	5603	3785	4823	5107	C（Energy production and conversion）	NAD(P)-dependent dehydrogenase, short-chain alcohol dehydrogenase family
COG0604	756	1002	694	956	1018	C（Energy production and conversion）	NADPH:quinone reductase or related Zn-dependent oxidoreductase
COG2165	1431	2502	1035	1159	1426	C（Energy production and conversion）	Type II secretory pathway, pseudopilin PulG
COG0683	951	1774	953	1025	1447	E（Amino acid transport and metabolism）	ABC-type branched-chain amino acid transport system, periplasmic component
COG0747	697	1686	667	744	1356	E（Amino acid transport and metabolism）	ABC-type transport system, periplasmic component
COG0174	277	644	509	538	593	E（Amino acid transport and metabolism）	Glutamine synthetase
COG1595	2464	3979	1889	2206	2488	K（Transcription）	DNA-directed RNA polymerase specialized sigma subunit, sigma24 family
COG0086	304	637	232	396	603	K（Transcription）	DNA-directed RNA polymerase, beta' subunit/160 kD subunit
COG0568	554	716	499	563	660	K（Transcription）	DNA-directed RNA polymerase, sigma subunit (sigma70/sigma32)
COG4771	1696	1946	1359	1807	1960	P（Inorganic ion transport and metabolism）	Outer membrane receptor for ferrienterochelin and colicins
COG2608	391	494	250	276	390	P（Inorganic ion transport and metabolism）	Copper chaperone CopZ
COG0605	293	411	259	279	401	P（Inorganic ion transport and metabolism）	Superoxide dismutase
COG0642	3392	4890	2778	3033	3216	T（Signal transduction mechanisms）	Signal transduction histidine kinase
COG0840	589	3763	847	1134	1744	T（Signal transduction mechanisms）	Methyl-accepting chemotaxis protein (MCP)
COG2199	689	3090	1075	1162	1701	T（Signal transduction mechanisms）	GGDEF domain, diguanylate cyclase (c-di-GMP synthetase) or its enzymatically inactive variants

Table S2. Functional genes exhibiting a stimulus-balance pattern influenced by domestic wastewater input and their abundances based on metagenomic analysis of the KEGG database.

KO	S-day28 （TPM）	S-day2（TPM）	Day0（TPM）	Q-day2（TPM）	Q-day28（TPM）	Classification by function	Module	Definition
K02406	134	449	242	276	319	Cell Motility	M02040	flagellin
K19611	168	486	203	235	326	Cell Motility	M02020	ferric enterobactin receptor
K03046	300	516	301	464	477	Signal Transduction	M03020	DNA-directed RNA polymerase subunit beta' [EC:2.7.7.6]
K03406	741	3679	791	976	1127	Signal Transduction	M02020	methyl-accepting chemotaxis protein
K21023	190	1120	281	414	504	Signal Transduction	M02025	diguanylate cyclase [EC:2.7.7.65]
K13924	187	839	371	480	616	Signal Transduction	M02020	two-component system, chemotaxis family, CheB/CheR fusion protein [EC:2.1.1.80 3.1.1.61]
K21449	1434	3831	2081	2943	3031	Signal Transduction	M02000	trimeric autotransporter adhesin
K11527	728	1816	889	1047	1100	Signal Transduction	M02022	two-component system, sensor histidine kinase and response regulator [EC:2.7.13.3]
K12132	805	5107	1828	2211	3157	Signal Transduction	M01001	serine/threonine-protein kinase [EC:2.7.11.1]
K01999	783	1622	793	893	1254	Membrane Transport	M02010	branched-chain amino acid transport system substrate-binding protein
K07795	1061	4812	998	1224	4472	Membrane Transport	M02020	putative tricarboxylic transport membrane protein
K19611	168	486	203	235	326	Membrane Transport	M02020	ferric enterobactin receptor
K02014	1573	2905	2058	2396	2536	Membrane Transport	M02000	iron complex outermembrane recepter protein
K02035	444	1406	643	701	1682	Membrane Transport	M02024	peptide/nickel transport system substrate-binding protein
K02004	859	1358	889	1111	1570	Membrane Transport	M02000	putative ABC transport system permease protein
K03286	712	1557	797	1163	1218	Membrane Transport	M02000	OmpA-OmpF porin, OOP family
K01915	429	785	525	626	691	Cell Growth and Death	M01100	glutamine synthetase [EC:6.3.1.2]
K00249	693	1241	764	952	1067	Cell Growth and Death	M00280	acyl-CoA dehydrogenase [EC:1.3.8.7]

Table S3. Functional genes exhibiting a stimulus-balance pattern influenced by domestic wastewater input and their expression levels, based on metatranscriptomic analysis of the COG database.

COG	S-day28（**TPM**）	S-day2（**TPM**）	Q-day2（**TPM**）	Q-day28（**TPM**）	Function	COG Description
COG2165	1076	4485	828	1644	C（Energy production and conversion）	Type II secretory pathway, pseudopilin PulG
COG0247	161	256	121	171	C（Energy production and conversion）	Fe-S cluster-containing oxidoreductase, includes glycolate oxidase subunit GlcF
COG0377	139	481	142	229	C（Energy production and conversion）	NADH:ubiquinone oxidoreductase 20 kD subunit (chain B) or related Fe-S oxidoreductase
COG0372	366	2228	230	1038	C（Energy production and conversion）	Citrate synthase
COG3181	94	1047	106	153	C（Energy production and conversion）	Tripartite-type tricarboxylate transporter, extracytoplasmic receptor component TctC
COG2010	1072	4699	1048	1421	C（Energy production and conversion）	Cytochrome c, mono- and diheme variants
COG1529	68	259	156	260	C（Energy production and conversion）	Aldehyde, CO or xanthine dehydrogenase, Mo-binding subunit
COG0747	426	2736	244	668	E（Amino acid transport and metabolism）	ABC-type transport system, periplasmic component
COG0624	198	302	95	121	E（Amino acid transport and metabolism）	Acetylornithine deacetylase/Succinyl-diaminopimelate desuccinylase or related deacylase
COG0665	89	742	50	92	E（Amino acid transport and metabolism）	Glycine/D-amino acid oxidase (deaminating)
COG0683	412	3695	560	1159	E（Amino acid transport and metabolism）	ABC-type branched-chain amino acid transport system, periplasmic component
COG1506	101	306	118	150	E（Amino acid transport and metabolism）	Dipeptidyl aminopeptidase/acylaminoacyl peptidase
COG0583	175	2337	126	208	K（Transcription）	DNA-binding transcriptional regulator, LysR family
COG1595	461	2961	1050	2284	K（Transcription）	DNA-directed RNA polymerase specialized sigma subunit, sigma24 family
COG1309	679	1859	177	408	K（Transcription）	DNA-binding protein, AcrR family, includes nucleoid occlusion protein SlmA
COG0086	908	1437	594	1015	K（Transcription）	DNA-directed RNA polymerase, beta' subunit/160 kD subunit
COG0568	863	1679	570	1455	K（Transcription）	DNA-directed RNA polymerase, sigma subunit (sigma70/sigma32)
COG4771	1688	5457	861	1629	P（Inorganic ion transport and metabolism）	Outer membrane receptor for ferrienterochelin and colicins
COG3119	28	358	235	342	P（Inorganic ion transport and metabolism）	Arylsulfatase A or related enzyme, AlkP superfamily
COG1629	309	1120	542	943	P（Inorganic ion transport and metabolism）	Outer membrane receptor protein, Fe transport
COG0642	526	1013	237	841	T（Signal transduction mechanisms）	Signal transduction histidine kinase
COG0515	172	1329	617	1407	T（Signal transduction mechanisms）	Serine/threonine protein kinase
COG2202	486	1826	485	1169	T（Signal transduction mechanisms）	PAS domain

Table S4. Functional genes exhibiting a stimulus-balance pattern influenced by domestic wastewater input and their expression levels, based on metatranscriptomic analysis of the KEGG database.

**KO**	S-day28（**TPM**）	S-day2（**TPM**）	Q-day2（**TPM**）	Q-day28（**TPM**）	**Classification by function**	**Module**	**KO_description**
K02651	944	8895	2112	4567	Cell Motility	M02020	pilus assembly protein Flp/PilA
K02406	988	5912	809	1303	Cell Motility	M02040	flagellin
K21449	1225	3746	1478	2417	Cell Motility	M02000	trimeric autotransporter adhesin
K04334	0	2559	2	20	Cell Motility	MO2026	major curlin subunit
K11527	437	751	156	201	Signal Transduction	M02022	two-component system, sensor histidine kinase and response regulator [EC:2.7.13.3]
K12132	486	1511	278	366	Signal Transduction	M01001	eukaryotic-like serine/threonine-protein kinase [EC:2.7.11.1]
K03406	366	1711	296	446	Signal Transduction	M03020	methyl-accepting chemotaxis protein
K03088	612	2923	920	2281	Signal Transduction	M03021	RNA polymerase sigma-70 factor, ECF subfamily
K06204	472	908	229	1888	Signal Transduction	M02026	RNA polymerase-binding transcription factor
K02014	702	1178	323	920	Membrane Transport	M02024	iron complex outermembrane recepter protein
K02035	218	4517	231	664	Membrane Transport	M02024	peptide/nickel transport system substrate-binding protein
K01999	1966	3638	372	499	Membrane Transport	M02010	branched-chain amino acid transport system substrate-binding protein
K02004	117	343	156	226	Membrane Transport	M02000	putative ABC transport system permease protein
K07795	166	1041	141	618	Membrane Transport	M02020	putative tricarboxylic transport membrane protein
K03286	1251	5896	832	1114	Membrane Transport	M02000	OmpA-OmpF porin, OOP family

Table S5. Differentially expressed key genes influenced by domestic wastewater input and their associated metabolic pathways.

Group	Module	Pathway	KO	KO_description
S-day2	M00542	O-Antigen repeat unit biosynthesis	K02851	UDP-GlcNAc:undecaprenyl-phosphate/decaprenyl-phosphate GlcNAc-1-phosphate transferase [EC:2.7.8.33 2.7.8.35]
S-day2	M00621	Dioxin degradation	K18366	acetaldehyde/propanal dehydrogenase [EC:1.2.1.10 1.2.1.87]
S-day2	M00621	Dioxin degradation	K02554	2-keto-4-pentenoate hydratase [EC:4.2.1.80]
S-day2	M00624	Polycyclic aromatic hydrocarbon degradation	K18068	phthalate 4,5-dioxygenase [EC:1.14.12.7]
S-day2	M00624	Polycyclic aromatic hydrocarbon degradation	K00449	protocatechuate 3,4-dioxygenase, beta subunit [EC:1.13.11.3]
S-day2	M00930	Caprolactam degradation	K01692	enoyl-CoA hydratase [EC:4.2.1.17]
S-day2	M00930	Caprolactam degradation	K01782	3-hydroxyacyl-CoA dehydrogenase / enoyl-CoA hydratase / 3-hydroxybutyryl-CoA epimerase [EC:1.1.1.35 4.2.1.17 5.1.2.3]
S-day2	M00561	Glycerolipid metabolism	K00655	1-acyl-sn-glycerol-3-phosphate acyltransferase [EC:2.3.1.51]
S-day2	M00561	Glycerolipid metabolism	K00128	aldehyde dehydrogenase (NAD+) [EC:1.2.1.3]
S-day2	M04122	Sulfur relay system	K04487	cysteine desulfurase [EC:2.8.1.7]
S-day2	M04122	Sulfur relay system	K00566	tRNA-uridine 2-sulfurtransferase [EC:2.8.1.13]
S-day2	M00040	Pentose and glucuronate interconversions	K00012	UDPglucose 6-dehydrogenase [EC:1.1.1.22]
S-day2	M00040	Pentose and glucuronate interconversions	K00963	UTP--glucose-1-phosphate uridylyltransferase [EC:2.7.7.9]
S-day2	M00450	Selenocompound metabolism	K00548	5-methyltetrahydrofolate--homocysteine methyltransferase [EC:2.1.1.13]
S-day2	M00450	Selenocompound metabolism	K01874	methionyl-tRNA synthetase [EC:6.1.1.10]
S-day2	M00791	Atrazine degradation	K01428	urease subunit alpha [EC:3.5.1.5]
S-day2	M00791	Atrazine degradation	K23359	biuret amidohydrolase [EC:3.5.1.84]
S-day28	M00230	Purine metabolism	K00525	ribonucleoside-diphosphate reductase alpha chain [EC:1.17.4.1]
S-day28	M00230	Purine metabolism	K01768	adenylate cyclase [EC:4.6.1.1]
S-day28	M00270	Cysteine and methionine metabolism	K01251	adenylate cyclase [EC:4.6.1.1]
S-day28	M00270	Cysteine and methionine metabolism	K00789	S-adenosylmethionine synthetase [EC:2.5.1.6]
S-day28	M00290	Valine, leucine and isoleucine biosynthesis	K01652	acetolactate synthase I/II/III large subunit [EC:2.2.1.6]
S-day28	M00290	Valine, leucine and isoleucine biosynthesis	K01649	2-isopropylmalate synthase [EC:2.3.3.13]
S-day28	M00300	Lysine biosynthesis	K00133	aspartate-semialdehyde dehydrogenase [EC:1.2.1.11]
S-day28	M00300	Lysine biosynthesis	K01714	4-hydroxy-tetrahydrodipicolinate synthase [EC:4.3.3.7]
S-day28	M00620	Pyruvate metabolism	K00138	aldehyde dehydrogenase [EC:1.2.1.-]
S-day28	M00620	Pyruvate metabolism	K01895	acetyl-CoA synthetase [EC:6.2.1.1]
S-day28	M00900	Terpenoid backbone biosynthesis	K00626	acetyl-CoA C-acetyltransferase [EC:2.3.1.9]
S-day28	M00900	Terpenoid backbone biosynthesis	K01662	1-deoxy-D-xylulose-5-phosphate synthase [EC:2.2.1.7]
S-day28	M00950	Isoquinoline alkaloid biosynthesis	K00812	aspartate aminotransferase [EC:2.6.1.1]
S-day28	M01210	2-Oxocarboxylic acid metabolism	K01647	citrate synthase [EC:2.3.3.1]
S-day28	M01210	2-Oxocarboxylic acid metabolism	K01652	acetolactate synthase I/II/III large subunit [EC:2.2.1.6]
